# The molecular components of the extracellular protein-degradation pathways of the ectomycorrhizal fungus *Paxillus involutus*

**DOI:** 10.1111/nph.12425

**Published:** 2013-07-31

**Authors:** Firoz Shah, Francois Rineau, Björn Canbäck, Tomas Johansson, Anders Tunlid

**Affiliations:** Department of Biology, Microbial Ecology Group, Lund UniversityEcology Building, SE-223 62, Lund, Sweden

**Keywords:** ectomycorrhizal (ECM) fungi, nitrogen assimilation, nitrogen catabolite repression, nitrogen transporters, *Paxillus involutus*, peptidases, transcriptional regulation

## Abstract

- Proteins contribute to a major part of the organic nitrogen (N) in forest soils. This N is mobilized and becomes available to trees as a result of the depolymerizing activities of symbiotic ectomycorrhizal fungi. The mechanisms by which these fungi depolymerize proteins and assimilate the released N are poorly characterized.
- Biochemical analysis and transcriptome profiling were performed to examine the proteolytic machinery and the uptake system of the ectomycorrhizal basidiomycete *Paxillus involutus* during the assimilation of organic N from various protein sources and extracts of organic matter.
- All substrates induced secretion of peptidase activity with an acidic pH optimum, mostly contributed by aspartic peptidases. The peptidase activity was transiently repressed by ammonium. Transcriptional analysis revealed a large number of extracellular endo- and exopeptidases. The expression levels of these peptidases were regulated in parallel with transporters and enzymes involved in the assimilation and metabolism of the released peptides and amino acids.
- For the first time the molecular components of the protein degradation pathways of an ectomycorrhizal fungus are described. The data suggest that the transcripts encoding these components are regulated in response to the chemical properties and the availability of the protein substrates.

## Introduction

A large fraction of nitrogen (N) in forest soils is present in organic form, including proteins but also other compounds such as amino sugars and heterocyclic N molecules (Nannipieri & Eldor, 2009). Moreover, the organic N compounds are associated with polyphenols, polysaccharides and other degradation products of plant and microbial polymers (Piccolo, 2001). Forest trees have a limited capacity to assimilate organic N (Näsholm *et al*., 2009), and they are generally thought to be dependent on depolymerizing activities by microorganisms such as ectomycorrhizal (ECM) fungal symbionts to access the organic N (Read *et al.*, 2004; Schimel & Bennett, 2004). This view was first supported by experiments demonstrating that plants of *Pinus contorta* associated with the ECM fungi *Paxillus involutus*, *Suillus bovinus* and *Rhizopogon roseolus* could grow on substrates supplemented with protein as a sole N source (Abuzinadah *et al.*, 1986). Since then, numerous studies have shown that ECM fungi can assimilate organic N from proteins as well as complex organic substrates such as litter material, pollen grains, and necromass of fungal mycelia and soil mesofauna (reviewed in Read *et al.*, 2004). In these experiments the N sources were exploited, with significant quantities of N being passed on to the host plant.

Despite the recognition that mobilization of N from organic matter by ECM fungi is of major importance for supporting forest trees with N, knowledge of the mechanism(s) involved is limited. Clearly, the assimilation of organic N by ECM fungi involves several steps, including the degradation of organic N polymers, assimilation of released mono- and oligomers, internal metabolism and transfer to the host plant (Chalot & Brun, 1998; Talbot & Treseder, 2010). Proteases are key enzymes involved in the extracellular degradation of proteins by fungi, and experiments have shown that the ability of *P. involutus* to capture N from plant-litter material is associated with increased protease activities in colonized material (Bending & Read, 1995). Furthermore, studies in pure culture systems using protein as a sole N source have shown that abilities to produce extracellular proteases is common among ECM fungi (Ramstedt & Söderhäll, 1983; Leake & Read, 1990; Zhu *et al.*, 1990; Maijala *et al.*, 1991; Nygren *et al.*, 2007). The proteolytic activity is typically expressed at an acidic pH (< 5.0) and is mainly inhibited by compounds active against aspartic proteases. Characterization of the extracellular proteolytic activity in *Hebeloma crustuliniforme* and *Amanita muscaria* showed that it is due to aspartic proteases (Zhu *et al.*, 1990; Nehls *et al.*, 2001). A cDNA presumably encoding one of the proteases (AmProt1) was also identified in *A. muscaria* (Nehls *et al.*, 2001). More recently, analysis of the genome and transcriptomes of *Laccaria bicolor* revealed that ECM fungi can express a large number of proteases and peptidases, not only including aspartic proteases but also members of the serine, metallo and cysteine classes of peptidases (Martin *et al.*, 2008). Furthermore, studies on amino acid and peptide transporters suggest that ECM fungi have a large capacity to assimilate the catabolites of extracellular proteases. Alhough only few transporters have fully been characterized (Nehls *et al.*, 1999; Wipf *et al.*, 2002; Benjdia *et al.*, 2006), *in-silico* analysis of the *L. bicolor* genome revealed that ECM fungi have a large gene repertoire for amino acid and oligopeptide transporters (Lucic *et al.*, 2008).

We have recently examined the molecular mechanisms by which *P. involutus* degrades polysaccharides and modifies polyphenols while assimilating organic N from plant-litter material. Data from spectroscopic and transcriptional analysis (Rineau *et al.*, 2012) provides evidence that the fungus converts such compounds using a radical-based biodegradation system involving Fenton chemistry that is similar to that of saprophytic brown-rot fungi. We also demonstrated that a Fenton-based degradation system is significantly stimulated by glucose whereas the effect of adding inorganic N is minute (Rineau *et al.*, 2013). However, the mechanism by which the fungus assimilates the organic N fraction of the soil and the fate of assimilated N in the fungus is not well understood.

In this study, we report on the proteolytic machinery expressed by *P. involutus* during the assimilation of organic N. Furthermore, to understand how this system is regulated depending on the properties of the N source, proteolytic activities were induced using a range of different organic N sources, including proteins, pollen and litter-material extracts. At a biochemical level, the extracellular protease activities induced by these substrates were similar. However, transcriptional analyses revealed differences of a large number of endo- and exopeptidases that contributed to this activity. The expression of transcripts encoding these enzymes was regulated in parallel with those of intracellular peptidases, amino acid and peptide transporters and enzymes involved in amino acid metabolism. This is a novel description of the molecular components involved in the assimilation and metabolism of N from protein substrates by ECM fungi.

## Materials and Methods

### Fungal strains and culture conditions

Cultures of *Paxillus involutus* (Batsch) Fr. (The American Type Culture Collection, ATCC 200175) were maintained aseptically on minimum Melin-Norkrans medium (MMN) agar plates containing glucose (2.5 g l^−1^), KH_2_PO_4_ (500 mg l^−1^), NH_4_Cl (200 mg l^−1^), MgSO_4_·7H_2_O (150 mg l^−1^), NaCl (25 mg l^−1^), CaCl_2_ (50 mg l^−1^), FeCl_3_·6H_2_O (12 mg l^−1^), thiamine-Cl (1 mg l^−1^) and agar (1.5%; pH 4.0). The fungus was grown on Petri dishes containing a glass-bead layer immersed in liquid MMN medium. A mycelia plug was cut from the margin of an actively growing mycelium (MMN agar) and transferred to the centre of the glass-bead plate. After 7 d of incubation (18°C, in the dark) when the colony reached a diameter of *c*. 4 cm and a biomass of 10 mg (DW), the medium was removed. The mycelium was washed in 10 ml autoclaved MilliQ (MQ) water, and 10 ml MMN medium without any N was added to induce an N-deprived mycelium. After 24 h, the mycelium was washed in MQ water and the liquid was replaced with 10 ml MMN media containing different protein substrates as sole N source or extracts of organic matter. Bovine serum albumin (BSA; 16% w/w N), gliadin (14% w/w N), and pollen (2–3% w/w N; all from Sigma-Aldrich) were the protein substrates used and were adjusted to a total N concentration of 53 mg l^−1^, which is equal to the total N concentration in MMN medium. The concentration of the organic matter extracts were adjusted according to Rineau *et al*. (2012). The organic matter extracts and MMN-protein medium were supplemented with glucose (final concentration 2.5 g l^−1^). The cultures were incubated for 7 d at 18°C in the dark.

### Preparation of organic matter extracts

Forest litter material was collected from the upper 10-cm soil layer in a 61-yr-old spruce stand growing in an N-poor site in central Sweden (pH 5.0). Maize compost was produced by cutting maize leaves into small pieces and composting them in a compost bin for 12 months. The litter and compost material were extracted with either cold or hot deionized water. Three organic extracts were generated: forest litter extracted with hot water (FH) and a maize compost extracted with cold (MC) or hot (MH) water. Particles were removed by sequential filtration and sterilized by filtration through 0.2 μm. Low molecular weight (MW) compounds were removed by ultrafiltration (cut-off 10 and 1 kDa, Amicon membranes; Merck Millipore). The same preparations of the FH, MH and MC extracts were used in all experiments. Further details are found in Rineau *et al*. (2012).

### Nitrogen starvation experiment

Mycelia grown for 7 d in the MMN medium were washed in sterile MQ water and then inoculated in MMN medium without N (i.e. omitting (NH_4_)_2_HPO_4_). The mycelia were harvested after 0, 2, 6, 24, 48, 72 and 96 h of incubation. Intracellular and soluble N was extracted in 300 μl by a methanol/methylene chloride/water solution (12/5/3, v/v/v; Botton & Chalot, 1991) in Eppendorf tubes and by grinding mycelia (3–5 mg, freeze-dried) with a plastic pestle. The fragmented mycelia were kept on ice and sonicated in an ultrasonic disintegrator (Vibra Cell), at 20–25 kHz for 2 min with a pause every 30 s. The extract was centrifuged for 20 min at 12 300 ***g***. The pellet was re-extracted twice, and the supernatants were pooled. The concentration of N was measured using a TOC/TN analyzer (Shimadzu, Kyoto, Japan) with a TNM-1 detector (Rineau *et al.*, 2012). The capacity of the fungus to express proteases following N deprivation was measured by adding the MMN medium containing BSA as a sole N source. The concentration of BSA was 342 mg l^−1^, which corresponds to the N content in the MMN medium (53 mg l^−1^). The protease activity was measured in culture filtrates as described later.

### Cell-wall bound proteolytic activity

In order to identify the levels and localization of the cell-bound peptidase activity, mycelium from 7-d-old cultures of *P. involutus* grown in MMN medium and using BSA as sole N source were used for preparation of cellular extracts. The mycelium was homogenized by grinding in liquid N_2_, resuspended in 1 ml 0.1 M Tris-HCl (pH 7.2) and sonicated (Mahadevan & Mahadkar, 1970). The mycelial slurry was then centrifuged at 16 000 ***g*** for 15 min at 4°C. The pelleted material was considered to represent extracellular cell-bound proteolytic activities whereas the supernatant was considered to represent soluble intracellular activities. The pellet was resuspended in the 1 ml of 0.1 M Tris-HCl buffer (pH 7.2) and used for enzymic measurements.

### Nitrogen repression experiments

*Paxillus involutus* was grown in MMN for 7 d, starved of N during 24 h and the medium was replaced with MMN containing BSA (342 mg l^−1^) as sole N source as described above. After 4 d, various concentrations of NH_4_Cl (0, 0.1, 0.5, 1.0, 5.0, 10, 20 mg l^−1^), KNO_3_ (0, 0.04, 0.2, 1.0, 5.0, 10, 20 mg l^−1^) and glutamic acid (0, 7.4, 14.7, 73.6, 147, 294, 736 mg l^−1^) were added to the medium to give a final concentration as mentioned within parantheses. The extracellular proteolytic activity was measured (as described below) after 0, 4, 14, 24 and 36 h, respectively.

### Enzyme activity measurements and characterization

The proteolytic activity was measured using a modified method described by Twining (1984). Fluorescein isothiocyanate coupled with BSA (FITC-BSA; Sigma-Aldrich) was used as substrate. The peptidase cleavage of the FITC-protein complex releases fluorescent compounds which are measured at neutral pH. Two-hundred microlitres of the samples were mixed with an equal volume of buffer (0.01 M Citrate-Cl, pH 3.0) and 10 μl FITC-BSA (2 mg ml^−1^ in citrate buffer) and the mixture was incubated at 37°C for 24 h. The reaction was terminated by adding 200 μl 10% TCA. After 1 h of incubation at room temperature, the sample was centrifuged at 16 000 ***g*** for 5 min. Then, 40 μl of the supernatant was mixed with 700 μl 0.4 M boric acid-NaOH buffer (pH 9.7) to neutralize the TCA-derived supernatant. Fluorescence was measured using a LS50B spectrofluorometer (Perkin-Elmer, Waltham, MA, USA) with excitation set to 490 nm and emission set to 525 nm. The protease activity is expressed in fluorescence units: one unit corresponds to the fluorescence produced by 0.33 ng ml^−1^ of trypsin during 24 h (Sigma-Aldrich). Protease inhibition was conducted by pre-incubating the culture filtrates with various protease inhibitors for 5 min at 37°C. The remaining enzyme activity was measured relative to an uninhibited control. The following inhibitors were used: pepstatin A (final concentration 0.01 mM), phenylmethylsulfonyl fluoride (PMSF; 1 mM), trans-epoxysuccinyl-l-leucylamido(4-guanidino)butane (E64; 0.01 mM) and EDTA (5 mM). To remove interfering compounds in the organic matter extracts (FH, MH and MC), the extracts were treated with polyvinylpolypyrrolidone (PVPP) before subjected to enzyme activity measurements (Rineau *et al.*, 2012).

Protease-activity visualization was performed using nondenaturing SDS-polyacrylamide gel electrophoresis (SDS-PAGE) containing 0.1% gelatin (Tunlid *et al.*, 1994). Media containing soil organic matter were not subjected to electrophoresis due to interferences by phenolics in visualization even after PVPP treatment. The protease activity in the culture filtrates were partially purified in batch by using an anion-exchange resin as previously described (Tunlid *et al.*, 1994). Briefly, Tris-HCl (pH 7.0) was added to a final concentration of 10 mM, followed by addition of a suspension of Q-Sepharose (GE Healthcare, Uppsala, Sweden). Measurements showed that during these conditions, 100% of the protease activity was bound to the resin. Bound proteins were eluted in 10 mM Tris-HCl (pH 7.5), 0.5 M NaCl and precipitated using ice-cold acetone followed by centrifugation at 16 000 ***g*** for 10 min. The pellet was dissolved in sample incubation buffer (Sigma-Aldrich) and applied onto a gel. After electrophoresis (100 V, 1.5 h), the gel was washed in 2.5% Triton X-100 in 0.01 mM citrate-Cl (pH 3.0) buffer for 1 h to remove excess of SDS. The gel was then incubated in 0.01 mM citrate-Cl (pH 3.0) buffer for 13–15 h at 37°C. After incubation, the gels were stained with Coomassie Blue G-250 for 1 h and destained in methanol : acetic acid : distilled water (30 : 10 : 60, v/v/v).

### RNA extraction and microarray

Fungal mycelia from growth on pollen, gliadin and BSA, as well as mycelia (from the Nitrogen repression experiment, see the two sections above) grown with and without ammonium for 14 and 36 h, respectively (+N14, −N14, +N36 and −N36, respectively), were collected. For each treatment there were three biological replicates (for each replicate mycelia collected from three Petri dishes) collected and immediately dropped into a clean mortar filled with liquid N_2_ and homogenized using a pestle. Total RNA was isolated using the RNeasy Plant Mini Kit (Qiagen) with the RLC buffer and the on-column DNase treatment according to the manufacturer. Total RNA was eluted in H_2_O and stored at −20°C until use. For quality assessments all samples were inspected using a RNA 6000 Nano kit on a 2100 Bioanalyzer (Agilent, Santa Clara, CA, USA).

A custom-designed microarray (12-plex 135K-oligonucleotide microarray, DesignID: 546871; NimbleGen/Roche, Basel, Switzerland) was used containing probes representing 12 214 transcripts (isotigs) obtained by 454/Roche DNA sequencing as well as Sanger sequencing (Applied BioSystems, Foster City, CA, USA) of a number of *P. involutus* transcriptomes collected during growth on various organic matter extracts and MMN medium. Each isotig was represented with up to 10 probes (60-mers) in a tiled design and the entire design is deposited at NCBI Gene Expression Omnibus (GEO; Edgar *et al.*, 2002; Ball *et al.*, 2004; accession GPL14950). Isotig sequences are also accessible at the *Paxillus* EST database: http://mbio-serv2.mbioekol.lu.se/Paxillus/Hybrid/ (to the given isotig ID add ‘paxillus_’ for searching) as well as at NCBI GenBank (accession SRA046093). The microarrays were hybridized as described in Supporting Information Notes S1. The raw images were bursted and processed using the NimbleScan software v2.5 (NimbleGen/Roche) and the built-in Robust Multichip Average (RMA) algorithm including quantile normalization for the purpose of removing the effects of systematic variation in the measured fluorescence intensities (Bolstad *et al.*, 2003; Irizarry *et al.*, 2003a,b). To this dataset we also introduced previously published data representing six biological replicates on the reference MMN medium (Rineau *et al.*, 2012) that are available at NCBI GEO (accessions GSM848412–GSM848414 and GSM848421–GSM848423), as well as three replicates each for FH (GSM848415–GSM848417), for MH (GSM848418–GSM848420) and for MC (GSM848424–GSM848426; all described in Rineau *et al.*, 2012) Normalized (log_2_-transformed) transcriptional values were brought into the Omics Explorer v2.2 (Qlucore, Lund, Sweden) for PCA and statistical analyses. The transcriptional data have been deposited at NCBI GEO and are accessible through the GEO SuperSeries accession number GSE47480.

### Bioinformatic analyses

Two different strategies were used for retrieving isotigs potentially encoding peptidases. First, isotigs containing Pfam domains that are related to enzymes of the MEROPS database (Studholme *et al.*, 2003; Rawlings *et al.*, 2012) were collected from the *P. involutus* EST database (http://mbio-serv2.mbioekol.lu.se/Paxillus/Hybrid/). The analysis generated a list of 277 isotigs that represented 49 unique Pfam families. Second, putative proteases/peptidases were also identified by searching the proteome of *P. involutus* (http://genome.jgi.doe.gov/Paxin1/Paxin1.home.html) against the MEROPS database with blastp. In total, 887 isotig sequences displayed sequence similarity (*E*-value threshold 1E-10) to protease sequences from fungi. The two listings were compiled and manually annotated to generate a listing of 312 isotigs encoding putative peptidases. Isotigs potentially encoding N-transporters and enzymes of amino acid metabolism were identified by using annotations for gene models of the *L. bicolor* genome (Lucic *et al.*, 2008; Rineau *et al.*, 2013).

Isotigs being significantly regulated during the assimilation of organic N were mapped to the corresponding gene model by using the Blast tool available at the JGI *Paxillus involutus* genome database (http://genome.jgi.doe.gov/Paxin1/Paxin1.home.html). Mapped gene models were manually inspected, and automatically selected best-gene models were manually modified if necessary. In the manuscript, the corresponding protein models are designated Pi:XXXX. Prediction of putative secretory signals was conducted using the SignalP v4.0 algorithm (Emanuelsson *et al.*, 2007). The probability (*P*) of observing the number of genes within a given category by chance was estimated using a hypergeometric distribution (Arvas *et al.*, 2007).

## Results

### Conditions for protease induction

**Fig1:**
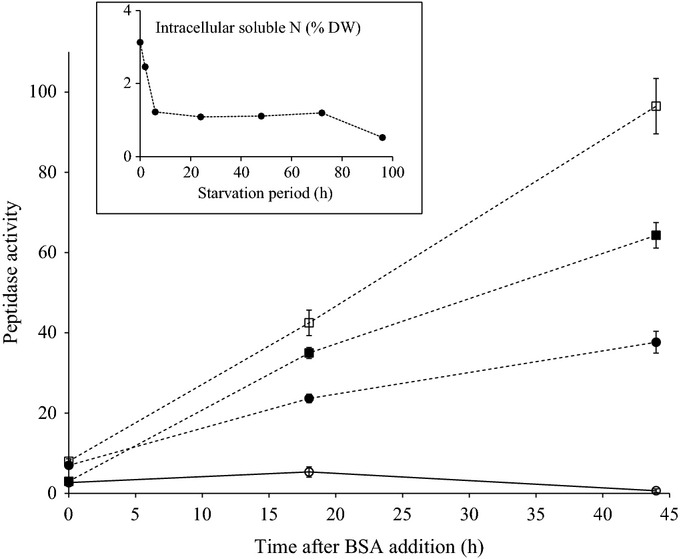
The effects of nitrogen (N) starvation on the expression of extracellular proteolytic activity by *Paxillus involutus*. The fungus was starved for 0 (continuous line, open circles), 2 (dotted line, closed circles), 24 (dotted line, open squares), or 96 (dotted line, closed squares) hours before the proteases were induced by adding BSA to the medium The extracellular peptidase activities are given in fluorescence units: one fluorescence unit is the fluorescence released by the activity of 0.33 ng ml^−1^ of trypsin over 24 h. Bars indicate ± SE (*n *=* *3). Inset: concentration of intracellular soluble N as a function of the starvation period at time points 0, 2, 6, 24, 48, 72 and 96 h.

In filamentous fungi, the utilization of organic N such as proteins is known to be regulated by two distinct signals: first, a global signal indicating N derepression, and second, pathway-specific signals that indicate the presence of the substrate (Marzluf, 1996). Initial experiments were conducted to establish the conditions for such signalling in *P. involutus* using BSA as a sole N source. Our experiment showed that *P. involutus* responded quickly and after 6 h of starvation, a marked decrease (to 75% of the level before starvation) in the content of intracellular, soluble N compounds was observed (Fig.1, inset). Adding BSA as an N source to the N-deprived mycelium induced extracellular protease activities (Fig.1). The extracellular peptidase activity was measured after starvation for 0, 2, 24 and 90 h. The highest level of extracellular protease activity was produced by the mycelium being starved for 24 h. The activity was lower when starved for shorter or longer periods of time. The reason for the reduced protease activity is not known, but it is well established that the catabolic capacity in *Saccharomyces cerevisiae* is declining during prolonged starvation periods (Albers *et al.*, 2007). No protease activity was detected when the fungus was grown in a medium lacking the inducing protein substrate (i.e. MMN medium without BSA). These results indicate that under N-starved conditions, proteins such as BSA can induce peptidase activity in *P. involutus*.

Excluding the intracellular fraction, nearly all (99%) of the protease activity induced by BSA was secreted into the culture filtrate. Thus, only 1% of the extracellular activity was associated with the mycelium cell wall (Fig. S1). In agreement with previous findings (Abuzinadah & Read, 1986; Zhu *et al.*, 1994; Nehls *et al.*, 2001), secretion of protease activity was observed in media at an acidic pH (< 5.0; not shown).

In the experiments following, a diverse set of organic N sources of different complexity and structure was used to induce proteolytic activity in N-starved mycelium (for 24 h) of *P. involutus*. Three different organic matter substrates were included: forest litter extracted with hot water (FH) and a maize compost extracted with cold (MC) or hot (MH) water, previously shown to be decomposed and used as N source for *P. involutus* (Rineau *et al.*, 2012). In addition, the expression of proteases was also examined in media containing pollen, gliadin or BSA. Earlier studies have shown that *P. involutus* can utilize these substrates as organic N sources (Abuzinadah & Read, 1986; Finlay *et al.*, 1992; Perez-Moreno & Read, 2001). The growth of *P. involutus* was enhanced by several of the N sources used, although there was a large variation in responses and growth could also be observed in media depleted for N (i.e. N-starvation conditions; Fig. S2).

### Characterization of extracellular protease activity

**Fig 2:**
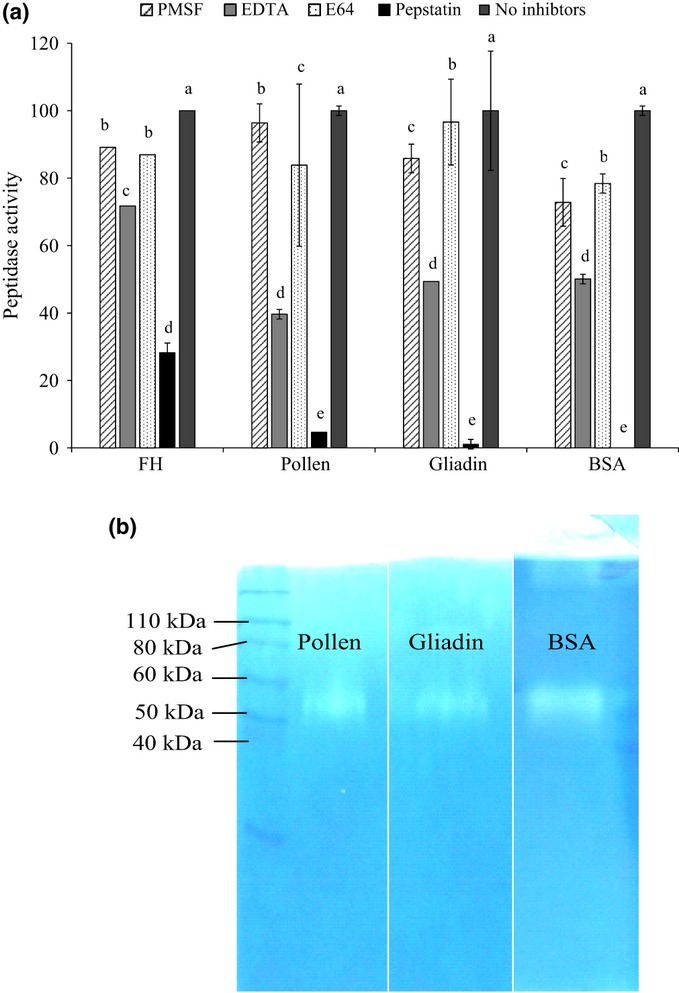
Characterization of the extracellular peptidase activity expressed by *Paxillus involutus* in forest litter extracted with hot water (FH), pollen, gliadin and BSA. (a) Effects of the protease inhibitors PMSF (active against serine proteases), EDTA (active against metalloproteases), E64 (active against cysteine proteases) and pepstatin (active against aspartic acid proteases) on the extracellular peptidase activities (in fluorescence units; one fluorescence unit is the fluorescence released by the activity of 0.33 ng ml^−1^ of trypsin over 24 h). The activity values are normalized, with ‘No inhibitors’ taken as 100% activity. The absolute peptidase activity corresponding to 100% was 46, 855, 713, 495 and 681 fluorescence units for the FH extracts, pollen, gliadin, BSA and casein samples, respectively. Bars indicate ± SE (*n *=* *3, except forest litter extract for which *n *=* *2). Effect of substrate: *P *<* *0.005; Effect of inhibitor: *P *<* *0.005 (two-way ANOVA). A Duncan test has been used to separate the significantly different means of the protease activity for the four different inhibitors and for each substrate. Average values that are significantly the same according to Duncan's test (confidence level = 0.95) share the same letter. (b) Substrate gel electrophoresis (zymogram) of culture filtrates of *P. involutus* when grown on pollen, gliadin or BSA as the nitrogen source. Novex Sharp Protein Standard (Invitrogen) with 12 bands ranging from 3.5 to 260 kDa was used as a molecular marker reference and some of those are indicated.

When grown in the media containing FH, pollen or protein (BSA, gliadin), *P. involutus* secreted a substantial amount of extracellular proteolytic activity (Fig.2a). Total peptidase activity declined in the order pollen > gliadin > BSA > FH extracts. The proteolytic activity in all substrates was significantly inhibited by the aspartic-protease inhibitor pepstatin (Fig.2a). Some inhibition was also obtained using EDTA, which indicates metallopeptidase activity. The cysteine inhibitor E64 and the serine-protease inhibitor PMSF had minute effects on the extracellular proteolytic activity. The pH optimum for this activity was at an acidic pH (*c*. 2.5–3.0; Fig. S3). Substrate gel-electrophoresis of the culture filtrates showed extracellular proteolytic activity in medium containing pollen, BSA and gliadin and was found in one single-electrophoretic band of *c*. 50 kDa (Fig.2b). The protease activity secreted by *P. involutus* when grown in media containing BSA, gliadin or pollen as a sole N source was similar to the extracellular protease activity induced by BSA in *H. crustuliniforme* (Zhu *et al.*, 1990) as well as in *A. muscaria* when grown in MMN medium (Nehls *et al.*, 2001). Thus, consistent with the main secreted protease activity in these fungi, the activity of *P. involutus* has a similar pH optimum (< pH 5.0), it is suppressed by the aspartic protease inhibitor pepstatin, and the activity can be focused by SDS-PAGE into a single band with an apparent MW of *c*. 40–50 kDa.

### Transcriptional responses during protease induction

**Fig 3:**
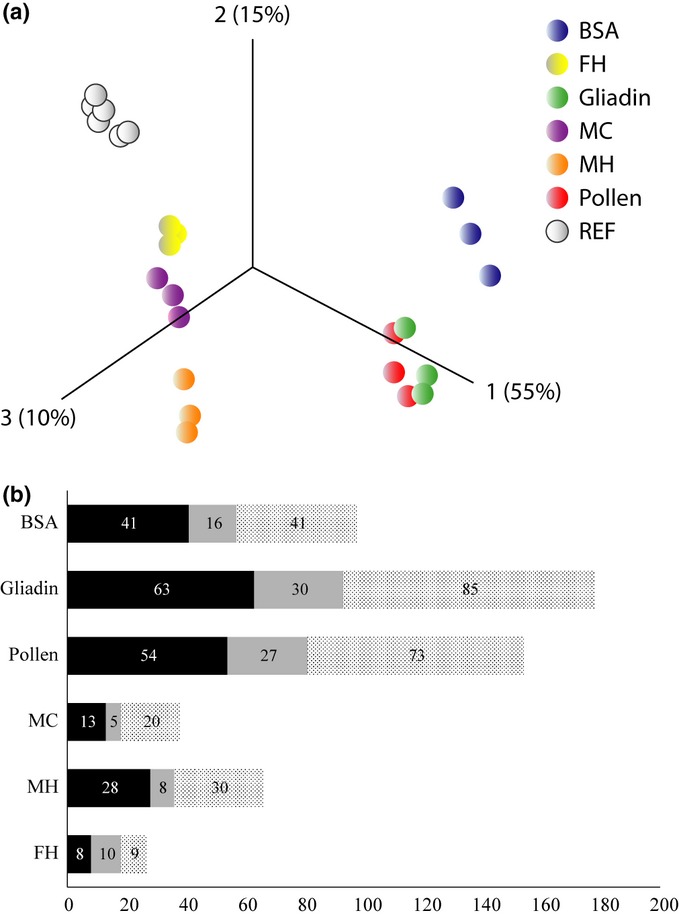
Transcriptional responses of *Paxillus involutus* during the assimilation of nitrogen (N) from complex organic matter media (forest litter extracted with hot water (FH), maize compost extracted with cold (MC) or hot (MH) water), pollen, gliadin or BSA. Minimum Melin Norkrans (MMN) is a mineral nutrient medium and is denoted as REF. (a) Principal component analysis (PCA) of the expression levels of 11 158 transcripts (isotigs; out of 12 214) that had a false discovery rate of *q *<* *0.01. *n *=* *3, except for MMN for which *n *=* *6. Each point in the PCA represents a replicate. (b) Number of transcripts that were upregulated at least two-fold (*q *<* *0.01) in the pairwise comparisons in the media containing organic matter or protein vs MMN and with annotations consistent with a role in the degradation, uptake and metabolism of proteins. Proteases, black bars; N transporters, grey bars; N, metabolism, stippled bars. The total number of isotigs annotated as proteases was 312, as N transporters 129 and as enzymes in N metabolism 292.

Microarray analysis showed that growth of the N-starved mycelium on various organic N sources induced a distinct difference in gene-expression patterns as compared to growth on MMN medium (Fig.3a). The transcriptional response was most pronounced in media containing gliadin or pollen, lower in BSA-containing medium and even lower in organic matter substrates: the number of at least two-fold upregulated transcripts (isotigs) in pairwise comparisons with MMN was 1464 in gliadin, 1367 in pollen, 852 in BSA, 619 in MH, 363 in MC and 140 in FH. Transcripts predicted to encode proteins/enzymes involved in the assimilation of organic N, including proteases, N transporters and enzymes of N metabolism, were significantly enriched (*P *<* *0.01; hypergeometric distribution) among the upregulated genes (Fig.3b).

**Fig 4:**
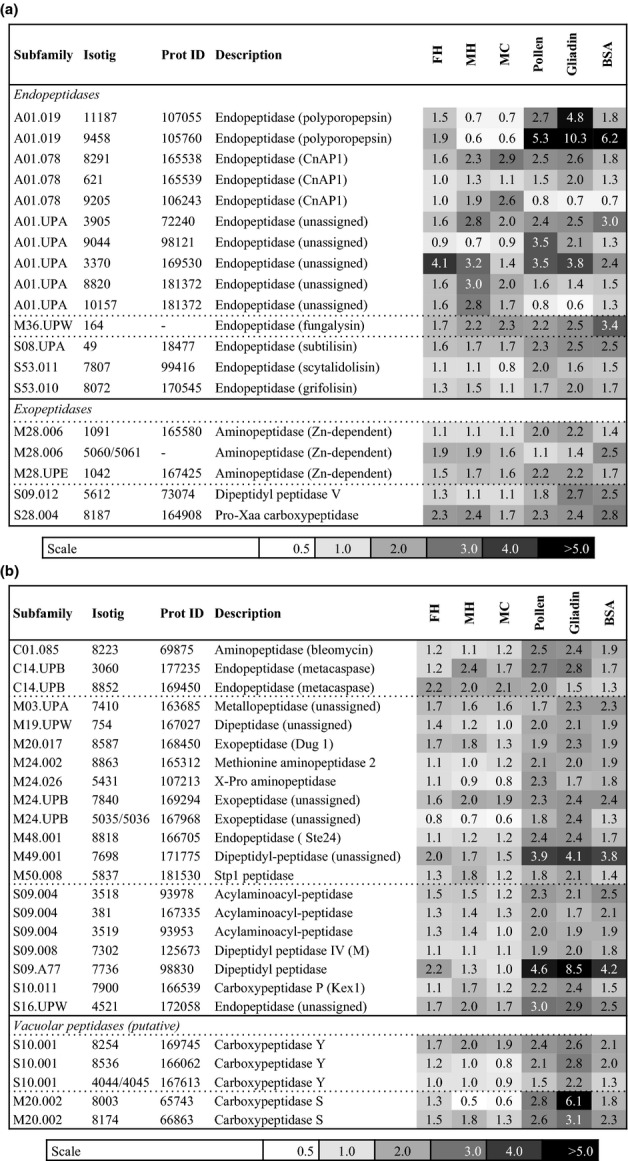
Regulation of genes predicted to encode peptidases in *Paxillus involutus* during the assimilation of organic nitrogen (N). Shown are the fold values of gene models that were upregulated more than two-fold (false discovery rate *q *<* *0.01) in at least one of the pairwise comparisons in media containing complex organic material or protein vs mineral nutrient (MMN) medium containing NH_4_Cl as the sole N source. Three different types of organic substrates were used: forest litter extracted with hot water (FH) and maize compost extracted with cold (MC) or hot (MH) water. The data presented average ratio of expression (*n *=* *3) as revealed by microarray analysis. ‘Subfamily’ refers to the ID of the family in the MEROPS peptidase database (Rawlings *et al.*, 2012); ‘Isotig’ refers to the transcript ID in the *Paxillus* EST database. ‘Prot ID’ refers to the predicted protein model in the *P. involutus* genome. (a) Genes predicted to encode secreted peptidases. (b) Genes predicted to encode nonsecreted (intracellular) peptidases.

*In-silico* analysis of the *P. involutus* EST database revealed the presence of 312 transcripts encoding putative proteases and peptidases. Using the MEROPS classification system, they included 26 aspartic, 73 cysteine, 132 metallo, 63 serine, 17 threonine families and one belonging to the glutamic-acid peptidase family. In total, 47 of these transcripts were found to be significantly upregulated by more than two-fold (false discovery rate *q *<* *0.01) in at least one of the pairwise comparisons in media containing extracts of complex organic material, pollen or protein vs inorganic N (MMN). The upregulated genes encoded a variety of secreted and nonsecreted endo- and exo-peptidases (Fig.4a,b). There was a large variation in the regulation of the peptidase transcripts depending on the organic N source.

**Fig 5:**
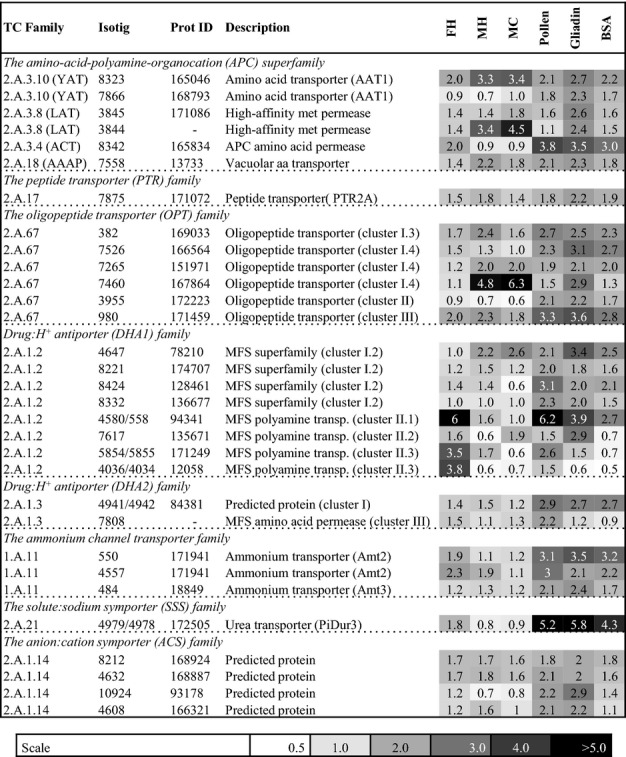
Regulation of gene models encoding nitrogen (N) transporters in *Paxillus involutus* during the assimilation of organic N. Shown are the fold values of 36 transcripts that were upregulated more than two-fold (false discovery rate *q *<* *0.01) in at least one of the pairwise comparisons in media containing complex organic material or protein vs mineral nutrient (MMN) medium containing NH_4_Cl as the sole N source. Three different types of organic substrates were used: forest litter extracted with hot water (FH), maize compost extracted with cold (MC) or hot (MH) water. The data presents the average ratio of expression (*n *=* *3). ‘TC Family’ is the classification system according to Saier (2000); ‘Isotig’ refers to the transcript ID in the *Paxillus* EST database; ‘Prot ID’ refers to the predicted protein model in the *P. involutus* genome (see Fig.4).

Based on manual annotations, we previously identified 129 transcripts encoding putative N transporters in the EST database of *P. involutus* (Rineau *et al.*, 2013). In total 36 of them were upregulated more than two-fold in at least one of the pairwise comparisons in the media containing organic N vs MMN. Apart from the YAAH/ATO family, upregulated genes were found in all the other families of N transporters identified in *P. involutus* (Fig.5).

In order to examine the intracellular metabolism of *P. involutus*, *in-silico* analyses identified 290 transcripts encoding such enzymes representing 144 unique EC numbers (Rineau *et al.*, 2013). Of these transcripts, 108 (77 ECs) were upregulated at least two-fold in at least one of the pairwise comparisons in media containing organic N vs ammonium (MMN). Genes being upregulated were found within all amino acid pathways (Table S1). The regulation of the enzymes in these pathways suggest that the amino acids that were released during protein degradation were actively metabolized and the N were utilized for the synthesis of other N-containing compounds including urea and polyamines (see the Discussion section).

### Nitrogen repression of the proteolytic activity

In order to examine whether the extracellular proteolytic activity of *P. involutus* was repressed in the presence of inorganic N and amino acids at concentrations found in forest soil extracts (Yu *et al.*, 2002; Andersson & Berggren, 2005; Werdin-Pfisterer *et al.*, 2009), ammonium (NH_4_Cl), nitrate (KNO_3_) or glutamic acid as principal N sources were added at increasing concentration to mycelium growing on medium containing BSA (Table1; Fig. S4). Partial repression of extracellular peptidase activity was obtained by the addition of NH_4_Cl at a concentration of ≥ 5 mg l^−1^. The effect was observed 14 h after adding the ammonium but the repression was not significant after 24 h. Considerably higher concentrations and longer incubation times were needed to detect repression by glutamic acid. No repression was observed by adding nitrate to the medium.

**Table 1:** Repression of proteolytic activity by ammonium and glutamic acid

	NH_4_Cl	Glutamic acid
Period of time before significant repression occurs (h)^a^	14	36
Lowest concentration inducing a significant repression (mg l^−1^)	5	147
Repression of protease activity at this concentration (SE with *n *=* *4)	80% (4 h)	45% (32 h)

*Paxillus involutus* was grown for 7 d on mineral nutrient medium (MMN), and starved of nitrogen (N) for 24 h before BSA was added. After another 4 d, NH_4_Cl or glutamic acid was added at increasing concentrations. No repression by nitrate observed and is excluded. The protease activity was analysed in the culture filtrates 0, 4, 14, 24 and 36 h after the addition of NH_4_Cl or glutamic acid (see Supporting Information Fig. S4). Values in parentheses indicate the time of incubation at which the indicated % repression was observed.

a ANOVA (*P *<* *0.001) of repression against no repression.

The transcriptional response of *P. involutus* during ammonium repression was minute. Only 20 transcripts out of the 12 214 isotigs represented on the microarray were significantly regulated (*P *<* *0.001) after incubation for 14 h in the presence of 5 mg l^−1^ NH_4_Cl as compared to the absence (Table S2). Furthermore, the magnitude of regulation was small (fold changes in the range 0.6 and 1.6). Only one of the regulated transcripts (isotig ID 8073) displayed sequence similarity to a protein with a possible role in the N assimilation (i.e. peptidases, N transporters and enzymes in N metabolism). This transcript displayed significant sequence similarity to a putative polyamine transporter of the DHA1 family. After 36 h of incubation, that is when repression of the activity was no longer detected, 17 transcripts were differentially expressed. None of them were found among those regulated after 14 h.

## Discussion

### Expression of extracellular peptidases

**Fig 6:**
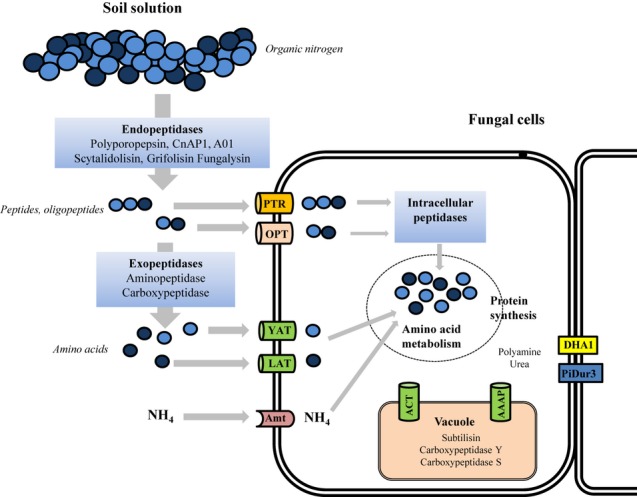
The molecular components of the protein degradation pathways of *Paxillus involutus*. Organic nitrogen (N) in soil solution is degraded by the combined action of endo- (polyporopepsin, CnAP1, A01 unassigned peptidases, fungalysin, scytalidoilsin and grifolisin) and exo-peptidases (aminopeptidases, carboxypeptidases) into peptides, amino acids and ammonium (see text for details). Peptides and oligopeptides assimilation is facilitated by Peptide transporter family (PTR) and Oligopeptide transporter family (OPT), amino acids are transported by Yeast amino acid transporter (YAT) and L-type amino acid transporter (LAT) while ammonium is internalized using Amt transporters. Intracellular peptidases further degrade oligopeptides and peptides while amino acids and ammonium are incorporated into the amino acid metabolic pathway. Intercellular transfer of urea and polyamine are facilitated by DHA1 (Drug:H^+^ antiporter) and PiDur3 (*P. involutus* degradation of urea transporter). Vacuolar peptidases are active on proteins transported through amino acid/choline transporter (ACT) and amino acid/auxin porter (AAAP).

Transcriptional analyses revealed a large number of peptidases and transporters expressed during the degradation of protein substrates by *P. involutus* (Fig.6). Considering extracellular peptidases, we identified nine genes encoding extracellular aspartate endopeptidases (Fig.4a). Based on sequence homology, they were classified into three MEROPS A1 subfamilies: the polyporopepsins, the CnAP1 peptidases and the A01 unassigned peptidases. Most upregulated in media containing pollen, gliadin and BSA were members of the polyporopepsin subfamily (described by Kobayashi, 2012). In the litter extracts, the most upregulated aspartate endopeptidases displayed close sequence homology to A01 unassigned peptidases, including AmProt1 from *A. muscaria* (Nehls *et al.*, 2001). Seven of the aspartate endopeptidases had a putative MW in the range 41–49 kDa. Two of them were predicted to encode significantly larger proteins with a MW of 75.9 kDa (Pi:181372) and 58.8 kDa (Pi:72240). Each sequence contained one predicted transmembrane helix, suggesting membrane-spanning properties. The activity of an atypical, larger aspartic endopeptidase (AmProt2; *c*. 100 kDa) has been reported in *A. muscaria* (Nehls *et al.*, 2001). In addition, there are two other MEROPS families of acidic extracellular endopeptidases identified: the serine-carboxyl peptidase and the glutamic peptidase families. Two genes encoding members in the sedolisin S53 family of the serine-carboxyl peptidases were significantly upregulated during protein degradation, including members of the grifolisin and the scytalidosin subfamilies (Suzuki *et al.*, 2005; Takahashi & Oda, 2008). The secreted protease activity of *P. involutus* was slightly suppressed by the metalloprotease inhibitor EDTA and the serine-protease inhibitor PMSF. Two members of these families were found among the upregulated transcripts, including a fungalysin (M36 subfamily) and a subtilisin (S8 subfamily; Graycar *et al.*, 2012; Kolattukudy & Sirakova, 2012).

It has been proposed that the ability of ECM fungi to utilize protein as N source depends on the synergistic action of secreted endo- and exopeptidases, because only amino acids and smaller peptides can be assimilated via membrane transporters (Chalot & Brun, 1998). In agreement with this suggestion, the transcriptional analysis revealed that *P. involutus* expressed both types of peptidases during digestion of proteins. Five genes predicted to encode extracellular exopeptidases were upregulated during degradation of the protein sources, including three aminopeptidases (M28 family), a dipeptidase (S9 family), and a carboxypeptidase (S28 family). Such exopeptidases are secreted by *A. fumigatus* during protein degradation at an acidic pH (Sriranganadane *et al.*, 2010). The upregulation of multiple endo- and exopeptidases suggests that *P. involutus* is an efficient organic N degrader.

### Assimilation of amino acids and peptides

During growth on protein-containing substrates, *P. involutus* expresses a large diversity of N transporters that are presumably involved in the assimilation of released amino acids and peptides (Fig.5). Among the highly upregulated transcripts were two members of the yeast amino acid transporter (YAT) family, and show a high sequence homology to the general amino acid transporter AAT1 of *A. muscaria* (Nehls *et al.*, 1999), which has been characterized as a high-affinity amino acid transporter with broad substrate specificity. Within the L-type amino acid transporter (LAT) family, two transcripts were upregulated that show significant sequence similarity to a high-affinity methionine permease in *L. bicolor* (Lucic *et al.*, 2008). Upregulated peptide transporters were found within two families: the peptide transporter (PTR) family and the oligopeptide (OPT) family. The PTR gene has close sequence homology to the high-affinity peptide transporter PTR2A of *Hebeloma cylindrosporum*, which is involved in the assimilation of di- and tripeptides (Benjdia *et al.*, 2006). Three phylogenetic clusters of OPT transporters have been identified in the genome of *L. bicolor* (Lucic *et al.*, 2008). The upregulated *P. involutus* transcripts were found in all three clusters implying active assimilation of peptides and amino acids.

### Intracellular metabolism and compartmentalization of assimilated nitrogen

Internalized peptides have to be hydrolysed into amino acids prior being metabolized or used as precursors in proteins synthesis. So far, no intracellular peptidases have been characterized in ECM fungi, but several candidates were found among upregulated transcripts in *P. involutus*, including dipeptidyl peptidases and unassigned endo- and exopeptidases (Fig.4b). The upregulated transcripts also represent peptidases with specific cellular functions, including stress response (bleomycin hydrolase, metacaspases; Kambouris *et al.*, 1992), the glutathione degradation pathway (Dug1; Kaur *et al.*, 2009), and protein processing (Stp1, Ste24, Map2; Li & Chang, 1995; Tam *et al.*, 2001; Bien *et al.*, 2009).

Studies particularly in yeasts and plants have shown that only a small fraction of the amino acids assimilated is metabolized and used for protein synthesis. The remaining fraction is metabolically inert and compartmentalized in vacuoles (Sekito *et al.*, 2008). Two of the upregulated transcripts in *P. involutus* showed high sequence homology to vacuolar amino acid transporters previously characterized in *S. cerevisiae*. A transporter (Pi:13733) of the amino acid/auxin porter (AAAP) family is a homologue to Avt4 that has a role in vacuolar amino acid export (Russnak *et al.*, 2001). The second putative vacuolar transporter (Pi:8342) is a member of the amino acid/choline transporter (ACT) family. This protein displays sequence homology to the *S. cerevisiae UGA4* gene, encoding a vacuolar permease involved in the transport and utilization of GABA and putrescine (Uemura *et al.*, 2004; Lucic *et al.*, 2008). Vacuoles also have important roles in degrading peptides and proteins. The molecular components of this pathway have been well characterized in *S. cerevisiae* (Li & Kane, 2009). Several homologues of these peptidases were found among upregulated transcripts in *P. involutus*, including carboxypeptidase Y and carboxypeptidase S (Knop *et al.*, 1993). The upregulated subtilisin shows close sequence homology to proteinase B, which suggests that it is localized to the vacuole. Together with the regulation of transcripts encoding vacuolar transporters, data suggest that the vacuoles have an active role in the storage and metabolism of the N compounds that are formed during protein degradation by *P. involutus*.

Glutamate/glutamine, alanine and aspartate/asparagine have been shown to be main sinks for N assimilated by ECM fungi (Finlay *et al.*, 1988; Morel *et al.*, 2005). Several of the most upregulated transcripts in *P. involutus* during the assimilation of organic N are representing enzymes involved in the amino acid metabolism, including glutamine synthetase (EC 6.3.1.2) and glutamate synthase (EC 1.4.1.13; Table S1; Morel *et al.*, 2006). The upregulation of glutamine-fructose-6-phosphate transaminase (EC 2.6.1.16), amidophosphoribosyltransferase (EC 2.4.2.14) and carbamoyl-phosphate synthase (EC 6.3.5.5) suggests that the N of glutamine and glutamate is utilized for the synthesis of amino sugars, purines and pyrimidines. Moreover, the upregulation of transcripts encoding aminotransferases, such as aspartate transaminase (EC 2.6.1.1) and alanine transaminase (EC 2.6.1.2) indicates an active transfer of amino groups between glutamate and other amino acids.

In a previous study it was shown that urea is one of the major N compounds found in the extraradical mycelium of *P. involutus* during mycorrhizal symbiosis with the *Betula pendula* (Morel *et al.*, 2005). Polyamines have also been found in high amounts in the mycelium of *P. involutus* grown axenically in synthetic medium (Fornalé *et al*., 1999). Both of these N compounds are derivatives of the urea cycle, and transcripts encoding for urea and polyamine transporters were upregulated in the extraradical mycelium of *P. involutus* (Morel *et al.*, 2005). PiDur3 was described as an urea import protein, involved in the transfer of urea in the extraradical mycelium (Morel *et al.*, 2005, 2008), whereas TPO3 was suggested to be a vacuolar transporter which allows long-distance translocation of N compounds along the mycelium, such as polyamines (Morel *et al.*, 2005). In agreement with these previous studies, we found several homologues for transporters and enzymes involved in the urea/polyamine metabolism that were significantly upregulated during protein degradation in *P. involutus*, including PiDur3, a putative polyamine transporter of the Drug:Hhar antiporter (DHA1) family which includes the TPO1-TPO4 polyamine transporters (Tomitori *et al.*, 1999; Albertsen *et al.*, 2003; Lucic *et al.*, 2008) and the UGA4 vacuolar transporter (Uemura *et al.*, 2004; Lucic *et al.*, 2008). In addition, our study also showed significant upregulation of transcripts encoding arginase, urease and ornithine decarboxylase, enzymes responsible for the synthesis of intracellular urea and polyamines (Table S1, Fig. S5). The synthesis of urea and polyamine is probably stimulated during C starvation. In accordance to our previous work (Rineau *et al.*, 2012), glucose is not detectable in the medium after 7 d of incubation with *P. involutus*. Carbon-rich compounds such as ornithine are formed by arginine degradation, which also generates urea (KEGG map00330; www.genome.jp/kegg/)), leading to high concentrations of urea and polyamines. Ornithine and urea are, however, degraded further in a catabolic arm of the urea cycle, to form polyamines such as putrescine/spermine and ammonium (Bago *et al.*, 2001). The expression of the metabolic enzymes in coordination with the expression of the urea and polyamine transporters suggests an active synthesis and transportation of urea and polyamine across the hyphae.

### Nitrogen catabolite repression

In agreement with findings in several other basidiomycetes, including both mycorrhizal and saprophytic species (Kalisz *et al.*, 1987; Leake & Read, 1991; Zhu *et al.*, 1994), the total extracellular proteolytic activity of *P. involutus* was mainly regulated by protein induction and only partially by ammonium repression. The experiments with the other basidiomycetes were conducted by growing the mycelia for several days in liquid media containing a protein inducer and ammonium at various concentrations. Similar experiments in *P. involutus* resulted in a variable responses ranging from stimulation to repression of proteolytic activity (not shown). To measure the nitrogen catabolite repression (NCR) response more precisely, ammonium, glutamic acid or nitrate were added to cultures at a time point when the mycelium was secreting high levels of proteolytic activity. Results demonstrated that the extracellular protease activity of *P. involutus* is repressible by ammonium (Fig. S4). The NCR has been studied in detail in filamentous ascomycetes (Cohen, 1973; Cohen & Drucker, 1977; Jarai & Buxton, 1994). In comparison with the response observed in these fungi, the repression in *P. involutus* was much less and it was restricted to a rather short period of time (< 24 h). In *Aspergillus nidulans*, ammonium, glutamine and glutamate have been identified as the signals for NCR (Margelis *et al.*, 2001). Nitrate is also used as an N source by *Aspergillus*, although it will not be utilized unless the cells are depleted for the favored compounds ammonium, glutamine or glutamate (Marzluf, 1996). In accordance with the role of these N sources in *Aspergillus*, NCR in *P. involutus* was induced by ammonium and glutamate, but not by nitrate. However, in contrast to ascomycetes (Marzluf, 1996), the repression of the protease activity in *P. involutus* did not correlate with a decreased level of transcripts encoding peptidases and amino acid/peptide transporters (Table S2). Accordingly, NCR of protease activity in *P. involutus* appears to be operating at the protein level.

The lessened NCR response agrees with a recent study showing that the addition of ammonium had relatively minor effects on the decomposition and assimilation of N by *P. involutus* from plant litter organic material (Rineau *et al.*, 2013). Moreover, the effects were only observed when glucose was added to the litter material. By contrast, numerous studies have shown that the decomposition of litter material by saprophytic fungi is repressed by high availability of N (Fenn & Kirk, 1981; Edwards *et al.*, 2011) and glucose (Aro *et al.*, 2005). However, the effects of N-availability on the expression of enzymes (including peptidases) involved in the degradation of litter material have only been considered for a small number of species. Hence, further studies are needed to reveal whether there are any consistent differences between saprophytic and ECM fungi in the regulation of such enzymes in response to more favourable N sources such as ammonium.

### Conclusions

The mobilization of protein N by ECM fungi is a biochemical process involving several stages including the breakdown of proteins, the uptake of the released mono- and oligomers, and the internal transformation of amino acids and peptides. Identifying the molecular components of this process is an important step towards identifying the mechanisms that control the use of organic N by mycorrhizal plants and how it may vary by species and environment. Our study shows that *P. involutus* expressed a large diversity of extracellular peptidases, in particular aspartate endopeptidases during the degradation of proteins. The relative expression levels of the genes encoding these enzymes varied depending on the protein source and the availability of the protein substrate (Fig.4a). A number of other fungi are known to express a battery of aspartate endopeptidases during the breakdown of protein substrates. For example, the secreted aspartic proteases (SAP) gene family of the human pathogen *Candida albicans* has 10 members and some of them are differentially regulated in response to specific environmental conditions (Naglik *et al.*, 2003). Moreover, heterologously expressed Sap peptidases can be clustered into three distinct groups based on their substrate specificity, indicating that they target different protein substrates during the infection (Aoki *et al.*, 2011). It remains to be determined whether the secreted aspartate peptidases of *P. involutus* differ in biochemical properties including substrate specificities.

The ability to assimilate N from range of different protein sources and environmental conditions will depend on the synergistic action of secreted endo- and exopeptidases and the expression of a matching set of N transporters. Indeed, the expression levels of N transporters in *P. involutus* varied extensively with the protein source (Fig.5). Further studies are however, needed to identify how the components of the extracellular peptidase machinery, the transporters and the internal metabolism are coordinately regulated in ECM fungi during the mobilization of different organic N sources available in forest soils (Fig.6).

## Supporting Information

**Fig. S1** Cellular localization of the peptidase activity expressed by *Paxillus involutus* during growth on a medium containing BSA as a N source. **Fig. S2** Growth response of *Paxillus involutus* when inoculated with substrates of different N sources, including organic matter extracts. **Fig. S3** pH optimum of the extracellular proteolytic activities secreted by *Paxillus involutus* during growth on pollen, gliadin or BSA. **Fig. S4** Nitrogen catabolite repression of the extracellular proteolytic activities of *Paxillus involutus*. **Fig. S5** Expression of enzymes in the arginine- and proline- metabolism pathway. **Table S1** The most upregulated transcripts encoding enzymes of N metabolism in *Paxillus involutus* during peptidase induction **Table S2** Transcripts regulated during N repression **Notes S1 **Additional methodological details for the microarray experiments.
