# Supplementary material for: The molecular components of the extracellular protein-degradation pathways of the ectomycorrhizal fungus Paxillus involutus
Source: New Phytol. 2013 Jul 31;200(3):875–87. doi: 10.1111/nph.12425 (PMC4282482; doi:10.1111/nph.12425)
Supplement: Supplementary file 1 [file nph0200-0875-SD1.pdf]

Supporting Information

Additional information for "The molecular components of the extracellular protein-degradation pathways of the ectomycorrhizal fungus *Paxillus involutus*"

Fig. S1 Cellular localization of the peptidase activity expressed by *Paxillus involutus* during growth on a medium containing BSA as a nitrogen source.

Fig. S2 Growth response of *Paxillus involutus* when inoculated with substrates of different nitrogen sources, including organic matter extracts.

Fig. S3 pH optimum of the extracellular proteolytic activities secreted by *Paxillus involutus* during growth on pollen, gliadin or BSA.

Fig. S4 Nitrogen catabolite repression of the extracellular proteolytic activities of *Paxillus involutus*.

Fig. S5 Expression of enzymes in the arginine- and proline- metabolism pathway.

Table S1 The most upregulated transcripts encoding enzymes of nitrogen metabolism in *Paxillus involutus* during peptidase induction.

Table S2 Transcripts regulated during nitrogen repression.

Note S1. Additional methodological details for the microarray experiments.

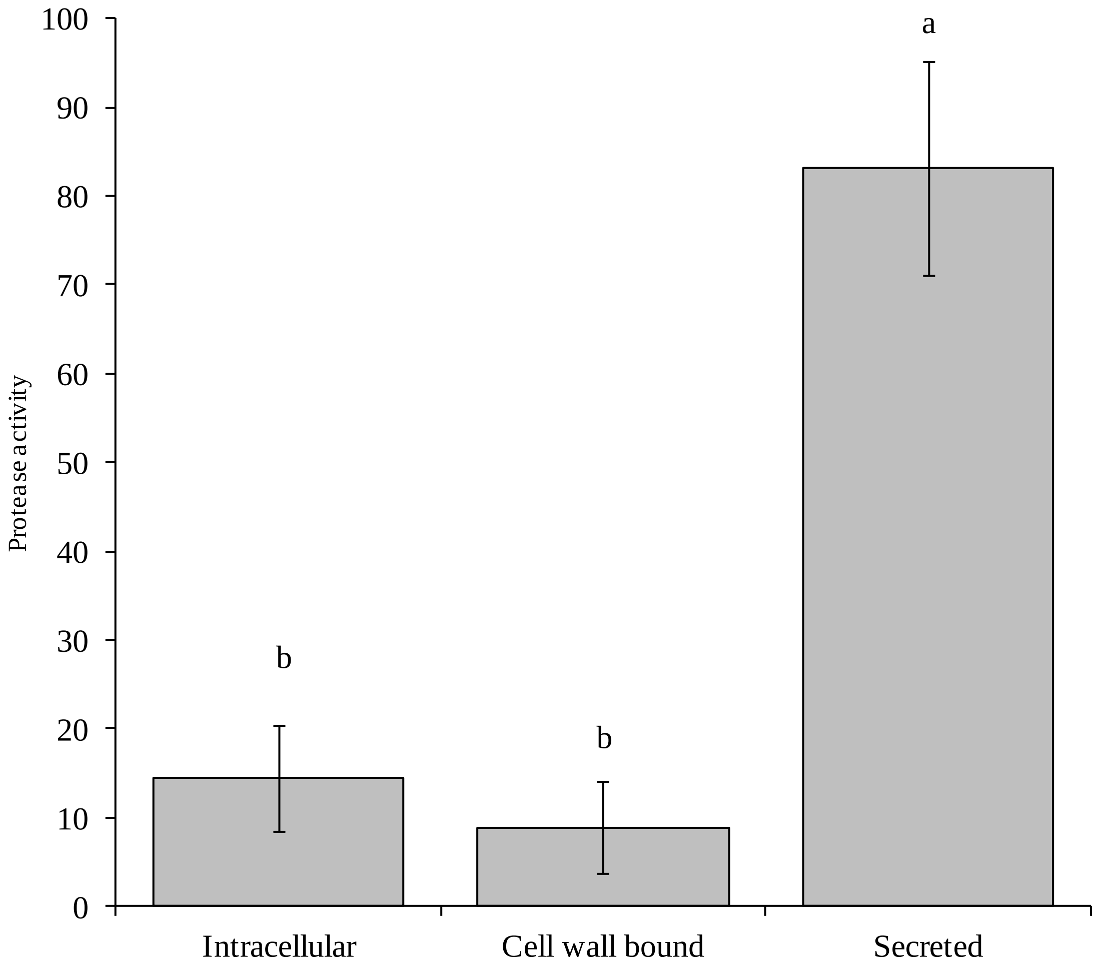

Fig. S1 Cellular localization of the peptidase activity expressed by *Paxillus involutus* during growth on a medium containing BSA as a nitrogen source. Bars indicate SE (n=6). Peptidase activity given in fluorescence units: one fluorescence unit is the intensity released by the activity of 0.33 ng ml^{-1} trypsin over 24 h.

A Duncan test has been used to separate the significantly different means of the protease activity of each fractions. Average values that are significantly the same according to Duncan's test (confidence level=0.95) share the same letter.

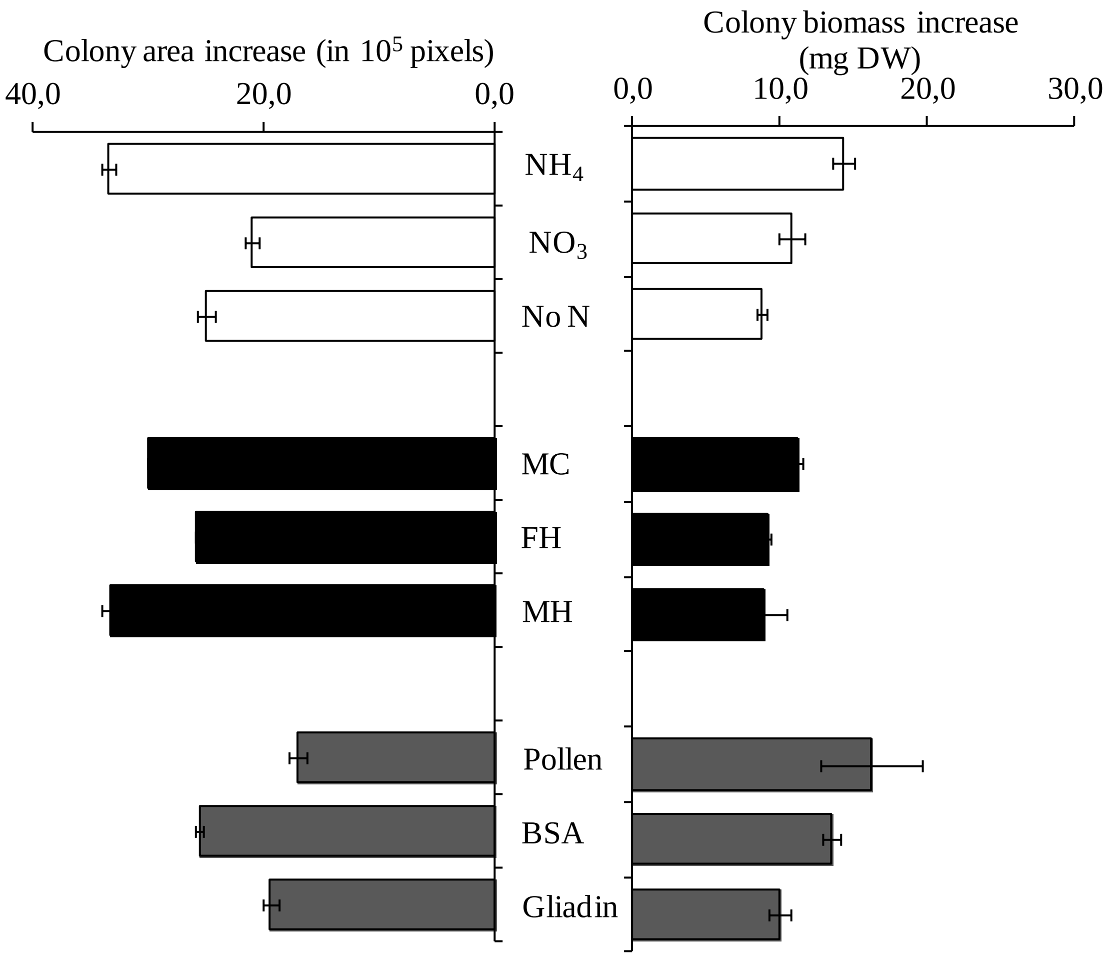

Fig. S2 Growth response of *Paxillus involutus* when inoculated with substrates of different nitrogen sources, including organic matter extracts. The mycelium was grown for 7 d on complete synthetic medium (MMN), starved for N during 24 h before adding medium containing the different N sources. The mycelium was inoculated for another 7 d before area growth and biomass were measured. FH indicates forest litter extracted with hot water (N content 100 mg l^{-1}); MH indicates maize compost extracted with hot water (N content 164 mg l^{-1}); MC indicates maize compost extracted with cold water (N content 170 mg l^{-1}). Bars indicate SE (n=3). DW, dry weight.

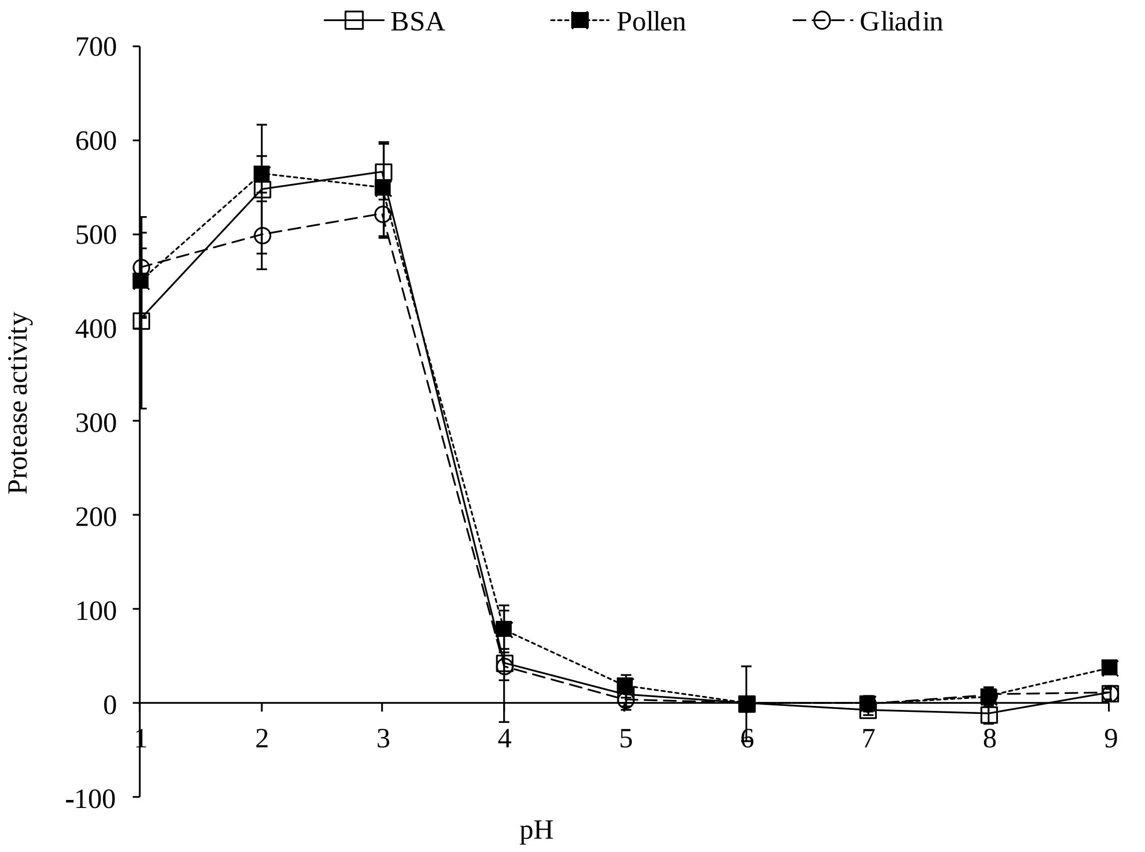

Fig. S3 pH optimum of the extracellular proteolytic activities secreted by *Paxillus involutus* during growth on pollen, gliadin and BSA. Bars indicate SE (n=3). Peptidase activity is given in fluorescence units: one fluorescence unit is the intensity released by the activity of 0.33 ng ml^{-1} trypsin over 24 h.

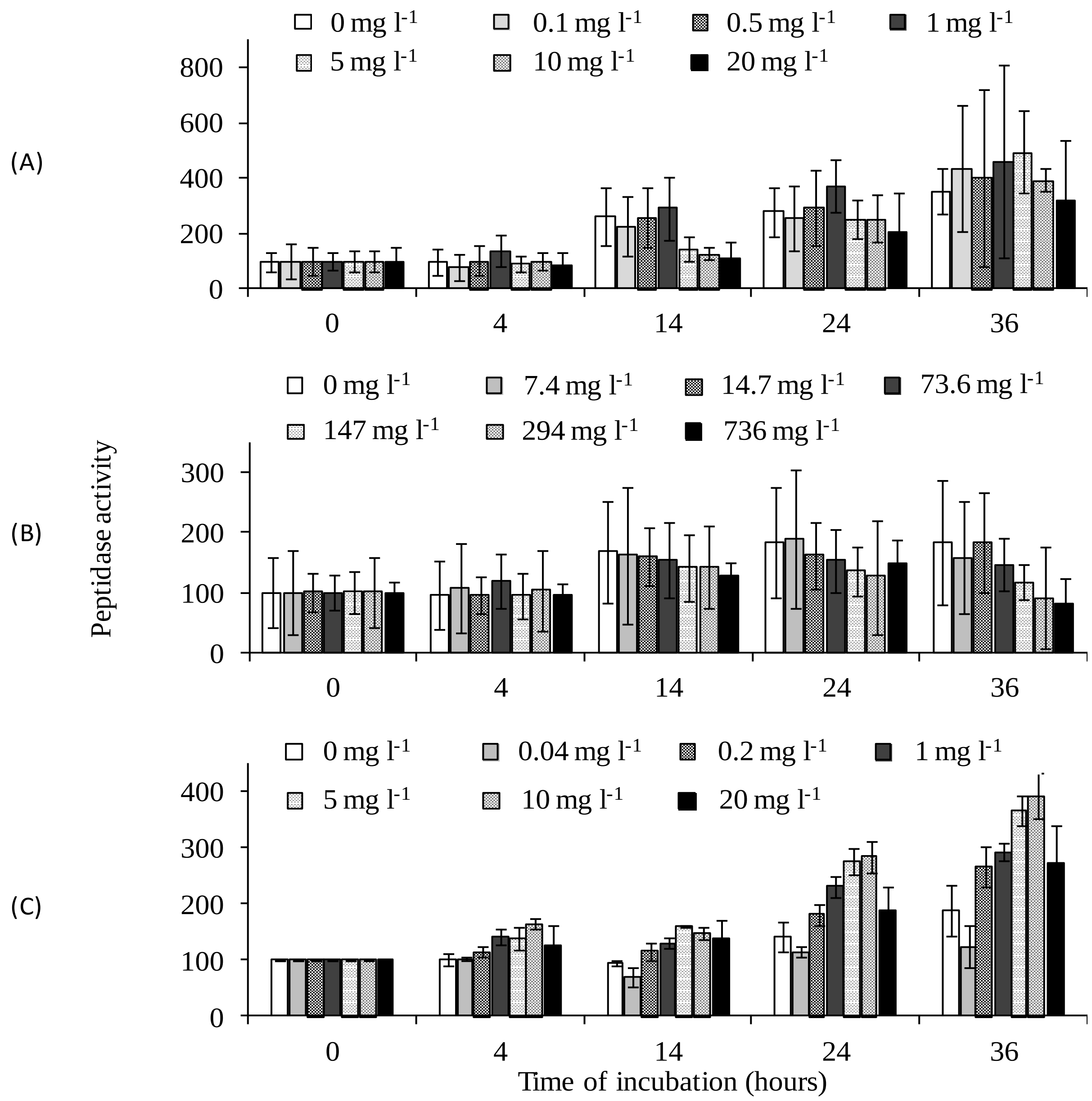

Fig. S4 Nitrogen catabolite repression of the extracellular peptidase activities of *Paxillus involutus*. The fungus was grown for 7 d on mineral nutrient medium (MMN) and starved for nitrogen for 24 h before BSA was added. After another 4 d, NH_4Cl (A), glutamic acid (B) or KNO_3 (C) was added at the indicated concentrations. The peptidase activity was analyzed in the culture filtrates 0, 4, 14, 24 and 36 h after the amendments. Peptidase activity is given in fluorescence units: one fluorescence unit is the intensity released by the activity of 0.33 ng ml^{-1} trypsin over 24 h. The values are normalized to 0 hour peptidase activity taken as 100 for all concentration for all treatments. Bars indicate SE (n=3).

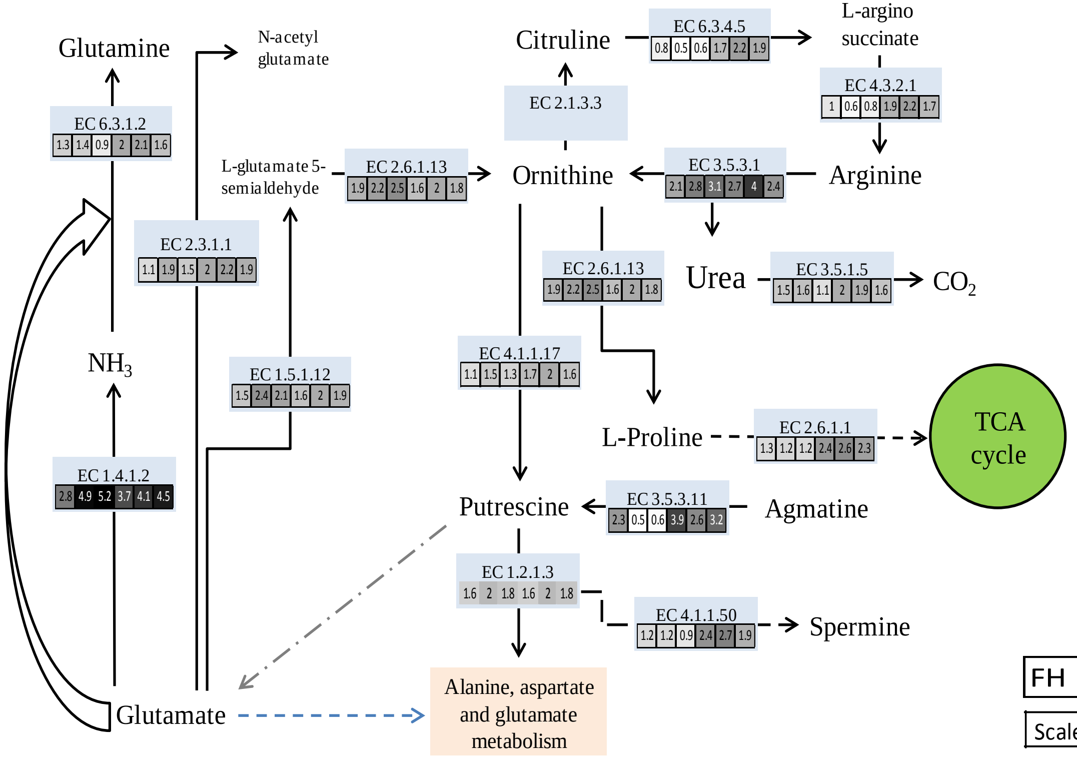

EC name	Description
1.2.1.3	Aldehyde dehydrogenase
1.4.1.2	Glutamate dehydrogenase
1.5.1.12	1-pyrroline-5-carboxylate dehydrogenase
2.3.1.1	Amino acid-N-acetyltransferase
2.6.1.1	Aspartate transaminase
2.6.1.13	Ornithine aminotransferase
3.5.1.5	Urease
3.5.3.1	Arginase
3.5.3.11	Agmatinase
4.1.1.17	Ornithine decarboxylase
4.1.1.50	Adenosylmethionine decarboxylase
4.3.2.1	Arginosuccinate lyase
6.3.1.2	Glutamate ammonia ligase
6.3.4.5	Argininosuccinate synthase

*Samples in sequence

FH	MH	MC	Pollen	Gliadin	BSA

Scale	0.5	1.0	2.0	3.0	4.0	>5.0

Fig. S5 Expression of enzymes in the arginine- and proline-metabolic pathway. Shown are the EC numbers and fold change values (color coded) of the transcripts encoding enzymes in *P. involutus* found to be upregulated more than two-fold (false discovery rate q<0.01) in at least one of the pairwise comparisons in media containing organic N sources versus mineral nutrient medium (MMN). The fold values are given from left to right (in boxes) for FH, MH, MC, pollen, gliadin and BSA compared with MMN. The values are average of multiple transcripts fold values, in cases there are more than one reporter representing the same EC number.

Table S1 The most upregulated transcripts encoding enzymes of nitrogen metabolism in P. involutus during peptidase induction. [a]

EC	Isotig ID	Descriptions	FH	MH	MC	POLLEN	GLIADIN	BSA
**Alanine, aspartate and glutamate metabolism**								
1.2.1.16	7381	Succinate-semialdehyde dehydrogenase	1.2	1.5	1.0	2.6	2.6	1.8
1.4.1.13	5283	Glutamate synthase (NADPH)	1.6	3.5	4.3	1.5	2.7	3.3
1.4.1.2	685	Glutamate dehydrogenase	2.8	4.9	5.2	3.7	4.1	4.5
1.5.1.12	1014	1-pyrroline-5-carboxylate dehydrogenase	1.5	2.4	2.1	1.6	2.0	1.9
2.4.2.14	7925	Amidophosphoribosyltransferase	1.2	1.6	1.2	2.8	2.4	1.5
2.6.1.1	6301	Aspartate transaminase	1.4	1.2	1.3	2.5	2.6	2.4
2.6.1.1	8978	Aspartate transaminase	1.2	1.2	1.2	2.3	2.5	2.2
2.6.1.16	7333	Glutamine---fructose-6-phosphate transaminase (isomerizing)	0.9	1.0	0.9	2.0	1.8	1.8
2.6.1.2	8275	Alanine transaminase	1.1	1.1	1.2	2.0	1.9	2.1
4.1.1.15	8502	Glutamate decarboxylase	0.7	0.7	0.7	1.5	2.4	0.8
4.3.2.1	2193	Argininosuccinate lyase	1.0	0.6	0.8	1.9	2.2	1.7
6.3.1.2	5599	Glutamate---ammonia ligase	1.3	1.4	0.9	2.0	2.1	1.6
6.3.4.5	169	Argininosuccinate synthase	0.8	0.5	0.6	1.7	2.2	1.9
6.3.5.5	4552	Carbamoyl-phosphate synthase (glutamine-hydrolysing)	1.4	1.8	1.6	1.7	1.5	2.0
**Arginine and proline metabolism**								
1.2.1.3	1023	Aldehyde dehydrogenase (NAD+)	1.6	1.6	1.6	1.5	1.8	2.0
1.2.1.3	7931	Aldehyde dehydrogenase (NAD+)	1.4	2.0	1.9	1.6	2.3	2.0
1.2.1.3	4595	Aldehyde dehydrogenase (NAD+)	1.8	2.5	1.9	1.7	2.0	1.4
1.4.1.2	685	Glutamate dehydrogenase	2.8	4.9	5.2	3.7	4.1	4.5
1.5.1.12	1014	1-pyrroline-5-carboxylate dehydrogenase	1.5	2.4	2.1	1.6	2.0	1.9
2.3.1.1	875	Amino-acid N-acetyltransferase	1.1	1.9	1.5	2.0	2.2	1.9
2.6.1.1	6301	Aspartate transaminase	1.4	1.2	1.3	2.5	2.6	2.4
2.6.1.1	8978	Aspartate transaminase	1.2	1.2	1.2	2.3	2.5	2.2
2.6.1.13	1010	Ornithine aminotransferase	1.9	2.2	2.5	1.6	2.0	1.8
3.5.1.5	4804	Urease	1.5	1.6	1.1	2.0	1.9	1.6
3.5.3.1	6190	Arginase	2.1	2.8	3.1	2.7	4.0	2.4
3.5.3.11	5071	Agmatinase	2.3	0.5	0.6	3.9	2.6	3.2
4.1.1.17	7590	Ornithine decarboxylase	1.1	1.5	1.3	1.7	2.0	1.6
4.1.1.50	8395	Adenosylmethionine decarboxylase	1.2	1.2	0.9	2.4	2.7	1.9
4.3.2.1	2193	Argininosuccinate lyase	1.0	0.6	0.8	1.9	2.2	1.7
6.3.1.2	5599	Glutamate---ammonia ligase	1.3	1.4	0.9	2.0	2.1	1.6
6.3.4.5	169	Argininosuccinate synthase	0.8	0.5	0.6	1.7	2.2	1.9
**Cysteine and methionine metabolism**								
1.2.1.11	925	Aspartate-semialdehyde dehydrogenase	1.2	1.2	1.2	2.2	2.2	1.7
2.1.1.14	4	5-methyltetrahydropteroyltriglutamate-homocysteine	2.4	2.7	2.8	3.2	3.5	4.5
2.5.1.-	8804	Protoheme IX farnesyltransferase	1.1	1.2	1.0	2.2	1.8	1.6
2.5.1.47	5953	Cysteine synthase	1.2	1.1	1.0	3.0	2.5	1.8
2.5.1.49	3886	O-acetylhomoserine aminocarboxypropyltransferase	0.9	1.1	1.1	1.5	2.1	1.6
2.5.1.6	1520	Methionine adenosyltransferase	1.3	1.4	1.5	2.1	2.4	2.1
2.6.1.-	10993	Aminotransferase class I and II family protein	1.2	1.0	0.9	2.0	2.1	1.2
2.6.1.1	6301	Aspartate transaminase	1.4	1.2	1.3	2.5	2.6	2.4
2.6.1.1	8978	Aspartate transaminase	1.2	1.2	1.2	2.3	2.5	2.2
3.3.1.1	228	Adenosylhomocysteinase	1.0	0.9	1.3	2.5	3.0	3.4
4.1.1.50	8395	Adenosylmethionine decarboxylase	1.2	1.2	0.9	2.4	2.7	1.9
4.3.1.17	798	Threonine ammonia-lyase	1.2	1.2	1.2	2.2	2.0	1.6
4.4.1.1	5541	Cystathionine gamma-lyase	1.6	1.9	1.6	2.5	3.0	2.5
**Glycine, serine and threonine metabolism**								

1.1.1.95	7277	Phosphoglycerate dehydrogenase	1.1	1.0	0.9	1.8	2.0	1.1
1.1.99.1	7732	Choline dehydrogenase	1.3	1.5	1.3	1.8	2.6	1.8
1.2.1.11	925	Aspartate-semialdehyde dehydrogenase	1.2	1.2	1.2	2.2	2.2	1.7
1.8.1.4	1030	Dihydrolipoyl dehydrogenase	1.1	1.5	1.4	2.3	2.5	2.3
2.1.2.1	8384	Glycine hydroxymethyltransferase	1.4	1.4	1.5	2.4	2.4	2.5
4.2.1.20	7692	Tryptophan synthase	1.5	1.7	1.4	2.3	2.2	1.8
4.2.3.1	5062	Threonine synthase	1.0	1.0	0.9	2.2	2.2	1.8
4.3.1.17	798	Threonine ammonia-lyase	1.2	1.2	1.2	2.2	2.0	1.6
4.4.1.1	5541	Cystathionine gamma-lyase	1.6	1.9	1.6	2.5	3.0	2.5
**Histidine metabolism**								
1.1.1.23	7386	Histidinol dehydrogenase	1.5	1.7	1.5	1.9	2.2	2.1
1.2.1.3	1023	Aldehyde dehydrogenase (NAD+)	1.6	1.6	1.6	1.5	1.8	2.0
1.2.1.3	7931	Aldehyde dehydrogenase (NAD+)	1.4	2.0	1.9	1.6	2.3	2.0
1.2.1.3	4595	Aldehyde dehydrogenase (NAD+)	1.8	2.5	1.9	1.7	2.0	1.4
1.2.1.5	8444	Aldehyde dehydrogenase [NAD(P)+]	0.9	0.9	1.7	1.4	2.6	1.0
2.1.1.-	6861	RNA methyltransferase	1.2	1.5	1.5	2.1	2.1	2.0
2.1.1.-	5783	shk1 kinase-binding protein 1	1.0	1.4	1.2	2.3	2.4	1.6
2.3.1.-	5907	Solute carrier family 33 (acetyl-CoA transporter)	1.4	1.7	1.3	2.1	2.2	1.9
2.3.1.-	8385	N-acetyltransferase ARD1 homolog	1.3	1.5	1.4	2.2	2.2	1.4
2.3.1.-	10259	MGC85493 protein	1.8	2.8	2.3	1.6	2.0	1.3
2.6.1.9	9407	Histidinol-phosphate transaminase	1.0	1.0	1.0	2.2	2.1	1.6
3.4.13.20	7099	Beta-Ala-His dipeptidase	1.3	1.6	1.3	1.9	1.7	2.0
3.4.13.20	8587	Beta-Ala-His dipeptidase	1.7	1.8	1.3	1.9	2.3	1.9
**Lysine biosynthesis**								
1.1.1.87	9390	Homoisocitrate dehydrogenase	0.9	1.0	0.9	2.0	2.1	1.6
1.2.1.11	925	Aspartate-semialdehyde dehydrogenase	1.2	1.2	1.2	2.2	2.2	1.7
1.2.1.31	4085	L-aminoadipate-semialdehyde dehydrogenase	0.9	0.8	1.0	2.0	1.7	1.5
2.6.1.-	10993	Aminotransferase class I and II family protein	1.2	1.0	0.9	2.0	2.1	1.2
3.5.1.-	1675	Sirtuin 2 (silent mating type information regulation)	1.2	1.6	1.6	2.4	3.0	1.9
**Lysine degradation**								
1.14.11.8	8277	Trimethyllysine dioxygenase	1.4	2.3	1.5	2.1	2.2	1.8
1.2.1.3	1023	Aldehyde dehydrogenase (NAD+)	1.6	1.6	1.6	1.5	1.8	2.0
1.2.1.3	7931	Aldehyde dehydrogenase (NAD+)	1.4	2.0	1.9	1.6	2.3	2.0
1.2.1.3	4595	Aldehyde dehydrogenase (NAD+)	1.8	2.5	1.9	1.7	2.0	1.4
1.2.1.31	4085	L-aminoadipate-semialdehyde dehydrogenase	0.9	0.8	1.0	2.0	1.7	1.5
1.2.4.2	4868	Oxoglutarate dehydrogenase (succinyl-transferring)	2.3	4.0	3.3	4.9	5.7	4.2
1.2.4.2	3431	Oxoglutarate dehydrogenase (succinyl-transferring)	1.4	2.5	1.8	0.7	0.8	1.3
1.3.99.7	8719	Glutaryl-CoA dehydrogenase	1.7	2.1	1.7	1.4	1.4	1.5
2.3.1.-	5907	Solute carrier family 33 (acetyl-CoA transporter)	1.4	1.7	1.3	2.1	2.2	1.9
2.3.1.-	8385	N-acetyltransferase ARD1 homolog	1.3	1.5	1.4	2.2	2.2	1.4
2.3.1.-	10259	MGC85493 protein	1.8	2.8	2.3	1.6	2.0	1.3
2.6.1.-	10993	Aminotransferase class I and II family protein	1.2	1.0	0.9	2.0	2.1	1.2
3.4.-.-	8187	Protease, serine	2.3	2.4	1.7	2.3	2.4	2.8
3.4.-.-	5431	X-pro aminopeptidase	1.1	0.9	0.8	2.3	1.7	1.8
4.2.1.17	1181	Enoyl-CoA hydratase	1.1	0.9	0.9	1.5	2.0	1.2
**Phenylalanine metabolism**								
1.2.1.5	8444	Aldehyde dehydrogenase [NAD(P)+]	0.9	0.9	1.7	1.4	2.6	1.0
2.3.1.-	5907	Solute carrier family 33 (acetyl-CoA transporter)	1.4	1.7	1.3	2.1	2.2	1.9
2.3.1.-	8385	N-acetyltransferase ARD1 homolog	1.3	1.5	1.4	2.2	2.2	1.4
2.3.1.-	10259	MGC85493 protein	1.8	2.8	2.3	1.6	2.0	1.3
2.6.1.1	6301	Aspartate transaminase	1.4	1.2	1.3	2.5	2.6	2.4
2.6.1.1	8978	Aspartate transaminase	1.2	1.2	1.2	2.3	2.5	2.2
2.6.1.9	9407	Histidinol-phosphate transaminase	1.0	1.0	1.0	2.2	2.1	1.6
**Phenylalanine, tyrosine and tryptophan biosynthesis**								
1.3.1.43	8778	Prephenate dehydrogenase (NADP+)	0.8	0.9	0.7	2.0	1.9	1.5
2.5.1.54	489	3-deoxy-7-phosphoheptulonate synthase	0.9	0.7	0.7	2.3	2.3	2.2
2.5.1.54	8851	3-deoxy-7-phosphoheptulonate synthase	1.5	1.7	1.5	2.1	1.9	1.5
2.6.1.9	9407	Histidinol-phosphate transaminase	1.0	1.0	1.0	2.2	2.1	1.6

4.2.1.20	7692	Tryptophan synthase	1.5	1.7	1.4	2.3	2.2	1.8
4.2.3.5	972	Chorismate synthase	1.0	1.0	1.0	2.0	2.1	1.9
**Tryptophan metabolism**								
1.11.1.6	924	Catalase	1.6	2.6	2.1	2.4	2.8	2.1
1.13.11.52	5918	Indoleamine 2,3-dioxygenase	1.0	1.1	1.0	2.1	1.6	1.2
1.14.-.-	4780	Cytochrome P450	1.3	2.3	1.5	2.1	1.9	2.2
1.14.14.1	8042	Unspecific monooxygenase	1.4	1.6	1.6	3.3	2.5	2.3
1.14.14.1	8011	Unspecific monooxygenase	1.3	1.1	1.2	1.8	2.2	1.7
1.14.14.1	8270	Unspecific monooxygenase	0.9	0.7	1.2	1.1	2.1	1.5
1.14.14.1	688	Unspecific monooxygenase	0.9	0.9	1.0	1.7	2.1	1.4
1.14.14.1	7958	Unspecific monooxygenase	0.8	0.8	0.8	1.8	2.0	1.2
1.14.14.1	5087	Unspecific monooxygenase	1.2	1.7	2.0	1.3	1.7	1.0
1.2.1.3	1023	Aldehyde dehydrogenase (NAD+)	1.6	1.6	1.6	1.5	1.8	2.0
1.2.1.3	7931	Aldehyde dehydrogenase (NAD+)	1.4	2.0	1.9	1.6	2.3	2.0
1.2.1.3	4595	Aldehyde dehydrogenase (NAD+)	1.8	2.5	1.9	1.7	2.0	1.4
1.2.4.2	4868	Oxoglutarate dehydrogenase (succinyl-transferring)	2.3	4.0	3.3	4.9	5.7	4.2
1.2.4.2	3431	Oxoglutarate dehydrogenase (succinyl-transferring)	1.4	2.5	1.8	0.7	0.8	1.3
1.3.99.7	8719	Glutaryl-CoA dehydrogenase	1.7	2.1	1.7	1.4	1.4	1.5
2.1.1.-	6861	RNA methyltransferase	1.2	1.5	1.5	2.1	2.1	2.0
2.1.1.-	5783	shk1 kinase-binding protein 1	1.0	1.4	1.2	2.3	2.4	1.6
2.5.1.-	8804	Protoheme IX farnesyltransferase	1.1	1.2	1.0	2.2	1.8	1.6
4.2.1.17	1181	Enoyl-CoA hydratase	1.1	0.9	0.9	1.5	2.0	1.2
6.3.2.-	7227	Ubiquitin-protein ligase	2.2	3.2	2.9	3.9	3.9	3.9
6.3.2.-	6908	CG15021 gene product from transcript	1.8	2.0	1.8	3.1	2.3	3.0
6.3.2.-	7307	CG15021 gene product from transcript	1.9	2.7	2.1	1.8	1.9	2.9
6.3.2.-	8805	CG15021 gene product from transcript	1.4	1.9	2.0	2.4	2.4	2.6
6.3.2.-	3783	CG15021 gene product from transcript	1.4	1.9	1.7	2.4	2.3	2.2
6.3.2.-	5893	CG15021 gene product from transcript	1.3	2.2	1.9	2.4	2.8	2.1
6.3.2.-	5382	CG15021 gene product from transcript	1.6	3.9	1.5	3.3	3.2	2.0
6.3.2.-	3495	CG15021 gene product from transcript	1.2	1.5	1.4	1.9	2.1	1.8
6.3.2.-	3818	CG15021 gene product from transcript	1.1	1.3	1.6	1.9	2.0	1.6
6.3.2.-	7885	E6-AP (UBE3A) carboxyl terminus) domain and RCC1 (CHC1)-like domain	1.0	1.2	1.0	2.0	2.2	1.6
6.3.2.-	7823	CG15021 gene product from transcript	1.2	1.3	1.2	2.5	2.1	1.6
6.3.2.-	7238	CG15021 gene product from transcript	1.3	2.1	1.5	1.2	1.3	1.4
6.3.2.-	891	CG15021 gene product from transcript	1.1	1.2	1.2	2.0	1.8	1.4
6.3.2.-	5594	checkpoint with forkhead and ring finger domains	1.2	1.4	1.2	2.1	2.0	1.2
6.3.2.-	11756	CG15021 gene product from transcript	1.2	1.6	1.1	2.2	1.9	1.2
6.3.2.-	9271	CG11734 gene product from transcript	1.2	2.0	1.3	1.5	1.5	1.1
**Tyrosine metabolism**								
1.1.1.1	9258	Alcohol dehydrogenase	1.3	0.9	1.0	4.6	3.1	3.6
1.2.1.16	7381	Succinate-semialdehyde dehydrogenase [NAD(P)+]	1.2	1.5	1.0	2.6	2.6	1.8
1.2.1.5	8444	Aldehyde dehydrogenase [NAD(P)+]	0.9	0.9	1.7	1.4	2.6	1.0
2.1.1.-	6861	RNA methyltransferase	1.2	1.5	1.5	2.1	2.1	2.0
2.1.1.-	5783	Kinase-binding protein 1	1.0	1.4	1.2	2.3	2.4	1.6
2.3.1.-	5907	Acetyl-CoA transporter	1.4	1.7	1.3	2.1	2.2	1.9
2.3.1.-	8385	N-acetyltransferase ARD1 homolog	1.3	1.5	1.4	2.2	2.2	1.4
2.3.1.-	10259	MGC85493 protein	1.8	2.8	2.3	1.6	2.0	1.3
2.6.1.1	6301	Aspartate transaminase	1.4	1.2	1.3	2.5	2.6	2.4
2.6.1.1	8978	Aspartate transaminase	1.2	1.2	1.2	2.3	2.5	2.2
2.6.1.9	9407	Histidinol-phosphate transaminase	1.0	1.0	1.0	2.2	2.1	1.6
**Valine, leucine and isoleucine biosynthesis**								
1.1.1.86	1711	Ketol-acid reductoisomerase	1.3	1.3	1.3	1.9	2.3	2.0
1.2.4.1	9054	Pyruvate dehydrogenase	1.3	1.5	1.2	2.3	2.4	2.3
2.2.1.6	3549	Acetolactate synthase	1.3	1.4	1.9	2.9	2.8	2.7
2.3.3.13	4290	2-isopropylmalate synthase	1.2	1.3	1.5	2.1	2.7	2.4
2.6.1.42	4634	Branched-chain-amino-acid transaminase	1.5	1.7	1.1	2.1	2.1	1.7
4.3.1.19	798	Threonine ammonia-lyase	1.2	1.2	1.2	2.2	2.0	1.6

6.1.1.4	7051	Leucine---tRNA ligase	1.7	2.1	2.1	3.2	3.5	4.6
6.1.1.9	5414	Valine---tRNA ligase	1.0	1.1	1.1	2.1	2.1	1.7
**Valine, leucine and isoleucine degradation**								
1.1.1.31	8864	3-hydroxyisobutyrate dehydrogenase	1.4	2.2	1.4	1.7	1.5	1.7
1.2.1.3	1023	Aldehyde dehydrogenase (NAD+)	1.6	1.6	1.6	1.5	1.8	2.0
1.2.1.3	7931	Aldehyde dehydrogenase (NAD+)	1.4	2.0	1.9	1.6	2.3	2.0
1.2.1.3	4595	Aldehyde dehydrogenase (NAD+)	1.8	2.5	1.9	1.7	2.0	1.4
1.2.4.4	1180	3-methyl-2-oxobutanoate dehydrogenase	2.2	4.5	3.3	1.6	2.4	1.5
1.3.99.-	8431	Acyl-Coenzyme A dehydrogenase	2.1	1.6	2.4	3.4	2.8	1.9
1.3.99.3	8829	Acyl-CoA dehydrogenase	1.4	1.6	1.4	2.5	2.2	1.6
1.3.99.3	11567	Acyl-CoA dehydrogenase	1.6	1.9	2.0	2.3	1.9	1.4
1.8.1.4	1030	Dihydrolipoyl dehydrogenase	1.1	1.5	1.4	2.3	2.5	2.3
2.3.1.16	8878	Acetyl-CoA C-acyltransferase	1.6	1.3	1.6	2.7	2.3	1.7
2.3.1.16	9650	Acetyl-CoA C-acyltransferase	1.5	2.5	1.3	1.9	2.1	1.2
2.3.3.10	712	Hydroxymethylglutaryl-CoA synthase	1.5	1.3	2.2	2.9	2.3	2.3
2.6.1.42	4634	Branched-chain-amino-acid transaminase	1.5	1.7	1.1	2.1	2.1	1.7
2.8.3.5	8369	3-oxoacid CoA-transferase	1.8	2.4	1.0	1.2	1.4	0.6
4.2.1.17	1181	Enoyl-CoA hydratase	1.1	0.9	0.9	1.5	2.0	1.2
6.4.1.3	8909	Propionyl-CoA carboxylase	1.6	2.9	2.4	1.3	1.7	1.3
6.4.1.4	7464	Methylcrotonoyl-CoA carboxylase	1.6	3.6	2.3	1.4	1.7	1.0

Scale	0.5	1.0	2.0	3.0	4.0	>5.0

[a] Shown are the expression profiles of transcripts that were manually annotated as encoding enzymes in amino acid metabolism of P. involutus and were upregulated more than two-fold (false discovery rate q<0.01) in at least one of the pairwise comparisons in media containing organic N sources versus mineral nutrient medium (MMN). The isotig ID refers to sequence information in the Paxillus EST database: http://mbio-serv2.mbioekol.lu.se/Paxillus/Hybrid/ (when searching, add “paxillus_” to the given isotig ID).

Table S2 Transcripts regulated during nitrogen repression.[a]

**Isotig ID** [b]	**Fold change**	**Uniprot homolog description**	**Pfam**
1248	1.6	Putative uncharacterized protein (*S. commune*) (D8PXY8)	
1325	1.5		
4457	1.5		
8447	1.3	Predicted protein (*L. bicolor*) (B0CU47)	WD 40 repeat (PF00400)
9736	1.3		
1329	1.3	Cytochrome c C1 (*L. bicolor*) (B0D4B4)	Cytochrome c family (PF00034)
811	1.2	Predicted protein (*L. bicolor*) (B0DG27)	Cytochrome c oxidase subunit IV (PF02936)
11949	1.2		
8455	1.1	Putative uncharacterized protein (*S. commune*) (D8Q805)	Phosphatidate cytidylyltransferase (PF01148)
6149	1.1	Oxidoreductase *(L. bicolor*) (B0DQT8)	Short-chain dehydrogenase (PF00106)
4091	0.9	TPR-containing protein Mql1 (*C. cinerea*) (A8N0U0)	Tetratricopeptide repeat (PF00515)
8073	0.9	Putative uncharacterized protein (*S. commune*) (D8PN04)	Major facilitator family (PF07690)
7049	0.9	Putative uncharacterized protein (*S. commune*) (D8PTE5)	Leucine-rich repeat (PF00560)
7754	0.9	Chitin synthase export chaperone (*C. cinerea*) (A8N0M3)	Chitin synthase III catalytic subunit (PF12271)
8339	0.8	Predicted protein (*L. bicolor*) (B0CU25)	DNA polymerase subunit Cdc27 (PF09507)
12814	0.8	Putative uncharacterized protein (*M. truncatula*) (B7FGY1)	Ubiquitin family (PF00240)
7968	0.8	Predicted protein (*L. bicolor*) (B0D7W2)	
6163	0.8	Putative uncharacterized protein *(S. commune*) (D8Q9T2)	BadF/BadG/BcrA/BcrD ATPase family (PF01869)
11320	0.8	Putative uncharacterized protein *(S. sclerotiorum*) (A7EAT8)	
5093	0.6	Predicted protein (*P. placenta*) (B8P175)	

[a] Shown are the fold values in the pairwise comparison of mycelium incubated for 14 hours in the presence (5 mg l-1) as compared with in the absence of NH4Cl.

[b] The isotig ID refers to sequence information in the Paxillus EST database: http://mbio-serv2.mbioekol.lu.se/Paxillus/Hybrid/ (when searching add “paxillus_” to the given isotig ID).

Note S1 Additional methodological details for the microarray experiments.

The microarray analyses were performed as single-label hybridizations. For each hybridization and each sample 10 μg of total RNA was used for cDNA synthesis using the SuperScript Double-Stranded cDNA Synthesis Kit (Invitrogen) according to the manufacturer. For quality assessments the produced cDNA was analyzed using a DNA 7500 kit on a 2100 Bioanalyzer (Agilent). For sample labeling the One-Color DNA Labeling Kit (Cy3) (NimbleGen/Roche) was used according to the manufacturer. After labeling, each sample received a Sample Tracking Control (NimbleGen/Roche) and hybridizations were immediately performed in a Hybridization System 4 (NimbleGen/Roche) for at least 16 h according to the manufacturer. The washing procedure was accordingly (NimbleGen/Roche) and the slides were finally scanned using an Agilent High-Resolution Microarray Scanner set at 20%PMT and 2 μm of resolution.
